# Pectinose induces cell cycle arrest in luminal A and triple-negative breast cancer cells by promoting autophagy through activation of the p38 MAPK signaling pathway

**DOI:** 10.1186/s12885-024-12293-8

**Published:** 2024-05-24

**Authors:** Shilong Yu, Zhaoyi Yue, Qilun Liu

**Affiliations:** General Hospital of Ningxia Medical University, Yinchuan, Ningxia 750004 China

**Keywords:** Breast cancer, Cell cycle arrest, Autophagy, MAPK signaling pathway

## Abstract

Breast cancer patients often have a poor prognosis largely due to lack of effective targeted therapy. It is now well established that monosaccharide enhances growth retardation and chemotherapy sensitivity in tumor cells. However, Pectinose whether has capability to restrict the proliferation of tumor cells remain unclear. Here, we report that Pectinose induced cytotoxicity is modulated by autophagy and p38 MAPK signaling pathway in breast cancer cell lines. The proliferation of cells was dramatically inhibited by Pectinose exposure in a dose-dependent manner, which was relevant to cell cycle arrest, as demonstrated by G2/M cell cycle restriction and ectopic expression of Cyclin A, Cyclin B, p21and p27. Mechanistically, we further identified that Pectinose is positively associated with autophagy and the activation of the p38 MAPK signaling in breast cancer. In contrast, 3-Ma or SB203580, the inhibitor of autophagy or p38 MAPK, reversed the efficacy of Pectinose suppressing on breast cancer cell lines proliferation and cell cycle process. Additionally, Pectinose in vivo treatment could significantly inhibit xenograft growth of breast cancer cells. Taken together, our findings were the first to reveal that Pectinose triggered cell cycle arrest by inducing autophagy through the activation of p38 MAPK signaling pathway in breast cancer cells,especially in luminal A and triple-negative breast cancer.

**Supplementary Information:**

The online version contains supplementary material available at 10.1186/s12885-024-12293-8.

## Introduction

Breast cancer is among the top-ranked malignancies in women, with characteristics including high incidence, heterogeneity, morbidity, mortality, and recurrence rate, as well as poor prognosis, which severely threatens human health [1]. Although adjuvant chemotherapy, targeted therapy, immunotherapy, and non-selective chemotherapy remain the cornerstones of treatment for breast cancer, they confer few benefits for patients with advanced stage disease [2–4]. Therefore, there is an urgent requirement to find additional or alternative drugs to improve therapeutic outcomes for patients with breast cancer.

Recently, regulatory relationships between monosaccharides and cancer cells have been established [5, 6]. And there is little evidence that Pectinose is also known as D-arabinose can inhibit the biological growth. For instance, D-arabinose can suppress the formation of biofilms originating from single or consortia bacteria on titanium discs by inhibiting the activity of the quorum sensing molecule, autoinducer 2 [7]. Furthermore, *Caenorhabditis elegans* growth is markedly inhibited by D-arabinose in a concentration dependent manner [8]. More importantly, Tanaka-Okamoto and colleagues found elevated levels of D-arabinose-containing free-glycans in the urine of cancer patients, indicating that D-arabinose may play a pivotal role in cancer development. Nonetheless, the regulatory functions and mechanism of D-arabinose influence the characteristic of cancer cells remain need to be elucidated.

Autophagy is an evolutionarily conserved catabolic process that parcels cytoplasmic proteins and damaged or senescent organelles into vesicles, which eventually fuse with lysosomes to form autophagy lysosomes for further degradation [9]. An increasing body of evidence has established the complex regulatory relationship between autophagy and cell cycle arrest in various cancer types [10, 11]. However, whether D-arabinose impaired growth by regulating cell cycle via induction of autophagy in breast cancer has not been studied.

In the present study, we demonstrated that breast cancer cell proliferation is sensitive to D-arabinose exposure, which induced cell cycle arrest. This phenomenon could be attributed to growth retardation mediated by induction of autophagy. Inhibition of autophagy counteracted the suppressive effect of D-arabinose on breast cancer cells. Furthermore, we found that activation of p38 signaling is responsible for the induction of autophagy by D-arabinose in breast cancer cells.

## Materials and methods

### Cell culture

The MCF-7, MDA-MB-231 and MCF10A cell lines were purchased from Procell (Procell, Wuhan, China), and were originally obtained from American Type Culture Collection. All cell lines were cultured in special culture medium purchased from Procell. Cells were maintained as monolayer cultures in a humidified atmosphere of 5% CO_2_ at 37 °C. When cultured cells reached 90% confluence, they were passaged after detachment using Recombinant Trypsin EDTA Solution (Biological Industries, Israel). All cell lines used were confirmed to be free of mycoplasma, and used for experiments when they entered an exponential growth stage.

### Assessment of cell viability and proliferation

MCF-7, MDA-MB-231 and MCF10A cells were seeded in triplicate in 96-well plates (1000 cells per well). After 24 h of adhesion culture, cells were treated with D-arabinose at different concentration. After 72 h incubation with different concentrations of D-arabinose, 10μL CCK8 solution (AR1160, Boster, China) was added to each well and incubated for 1-4 h. Absorbance of each well was measured at 450 nm using a microplate reader. The percentage of viable cells was determined for each well using the following equation: Percentage cell viability = (OD sample—OD blank)/ (OD control—OD blank) × 100%.

### Colony formation assay

MCF-7 and MDA-MB-231 cells were seeded in 6-well plates at 2000 cells per well. After incubation overnight, cells were treated with various concentration of D-arabinose for a further two weeks. Then, plates were washed three times with cold PBS, fixed with 4% paraformaldehyde at room temperature for 15 min, and stained with 0.1% crystal violet for 20 min. After washing with distilled water until the background became clear, images were photographed using a digital camera. Numbers of colonies in each well were quantified by microscopy.

### Cell cycle analysis

Adherent breast cancer cells were exposed to 50 mM D-arabinose for 24 h. Cells were then suspended using 0.25% trypsin–EDTA and washed with ice-cold PBS. Then, control and treated group cells were fixed in chilled 75% ethyl alcohol for 1 h at 4 °C before being transferred to -20 °C until they were required for analysis. After washing three times with PBS, cells were suspended in 0.1 mg/ml propidium iodide at 37 °C for 30 min in the dark. The distribution of cells in cell cycle phases was assessed by flow cytometry, and the number of cells at each event, namely, G0/G1, S, and G2/M phases, determined in control and D-arabinose-treated cells.

### Western blot assays

Cells or tissue derived from tumor-bearing mice were washed three times with cold PBS and lysed in cold RIPA buffer supplemented with protease inhibitor cocktail (Thermo Fisher Scientific, USA). Lysates were centrifuged at 12,000 × g at 4 °C for 5 min, and isolated protein samples mixed with loading buffer and boiled at 100 °C for 5 min. Total protein concentrations were measured using a bicinchoninic acid protein assay kit (Beyotime, Jiangsu, China). Equivalent amounts of protein samples were separated by SDS-PAGE and transferred to PVDF membranes (Millipore, Sigma, USA). After blocking with 5% skimmed milk, membranes were probed with the following antibodies: GLUT1 (1:1000, Serbicebio), Cyclin B1/2 (1:1000, Zenbio), CDK1 (1:1000, Zenbio), p21(1:1000, Proteintech), p27(1:1000, Proteintech), Atg5 (1:1000, Proteintech), LC3-I/II (1:500, Proteintech), ERK1/2 (1:1000, Ptmbio), p-ERK/2 (1:1000, Ptmbio), p38 (1:1000, Wanlcibio), p-p38 (1:1000, Wanlcibio), JNK (1:1000, Proteintech), p-JNK (1:1000, Proteintech), and β-Tubulin (1:1000, Solarbio). After incubation with corresponding horseradish peroxidase-conjugated secondary antibodies for 1 h at room temperature and extensive washes with TBST, immunoreactive proteins were visualized using enhanced chemiluminescence detection reagents (Sigma). Finally, images of the membranes were captured using a gel imaging system, analyzed with Image J 1.4.3 software (Bethesda, MD, USA), and normalized to β-Tubulin levels, followed by calculations of relative ratios to controls.

### Lentivirus construction and infection

Lentiviruses expressing short hairpin RNA (shRNA) specific for Atg5 were designed and synthesized by Hanbio Tech (Shanghai, China). Target nucleotide sequences were as follows: Atg5-shRNA, 5′-CCTGAACAGAATCATCCTTAA-3′ (sh-Atg5); scrambled control-shRNA, 5′-CCTAAGGTTAAGTCGCCCTCG-3′ (sh-NC). A lentivirus multiplicity of infection (MOI) of 20 was used for infection of MCF-7 and MDA-MB-231 cells.

### Autophagy flux analysis

Cells plated on coverslips in 24-well plates were infected with adenoviral vector expressing mRFP-GFP-LC3 (Hanbio Tech, Shanghai, China) at a MOI of 20 for 24 h. After D-arabinose treatment, cells were fixed with 4% ice-cold paraformaldehyde (Beyotime, Jiangsu, China) for 10 min, embedded in fluorescent mounting medium with DAPI (4,6-diamidino-2-phenylindole), and autophagic flux analyzed by assessing the number of GFP and mRFP puncta visible under confocal microscopy (TCS SP5, Leica Microsystems, Wetzlar, Germany).

### Mouse xenograft model

Four-week-old BALB/c female nude mice were purchased from Charles River (Beijing, China). All animals were acclimated under standard laboratory conditions (ventilated room, 25 °C ± 1 °C, 60% ± 5% humidity, 12 h light/dark cycle) and had free access to standard water and food. All procedures were conducted in accordance with the “International Council for Laboratory Animal Science (ICLAS)” and approved by the Laboratory Animal Ethics and Welfare Committee of the Laboratory Animal Center of Ningxia Medical University (IACU-NYLAC-2021-035). Mice were adapted to the breeding environment for three days before the experiment. A total of 2 × 10^6^ MDA-MB-231 cells were suspended in 100 μL PBS with Matrigel and injected into the breast fat pads of nude mice, then tumor growth observed. When tumors were visible to the naked eye, tumor-bearing mice were randomly divided into two groups (control and experimental) of 5 mice each. An aqueous solution containing 50% (w/v) D-arabinose was administered to mice by gavage 200μL/every day. Tumor volume was measured twice per week using vernier calipers, and the tumor volume calculated according to the formula: Volume = (Length × (Width)2)/2. Mouse activity, weight, food and water intake, and other basic indicators were observed. After 4 weeks, mice were executed by cervical dislocation, where the left thumb and forefinger pressed down hard on the head of the mouse, and the right hand grasped the tail and pulled it back hard, and the mouse died immediately, causing a painless death. The tumors were collected, weighed, and photographed. Then, tumor tissues were divided into two parts: one of which was stored in liquid nitrogen for total protein extraction and the other fixed in 4% paraformaldehyde for subsequent pathological examination.

### Histopathology and Immunohistochemistry

Mouse tissue samples were fixed in paraformaldehyde and embedded in paraffin. Tumor specimens were stained with hematoxylin and eosin after cutting into 5 µm sections. For immunohistochemistry assays, slides were incubated with Ki67 after blocking with 5% BSA. The next day, samples were incubated with secondary antibodies at 37 °C for 1 h, followed by treatment with DAB chromogen. Images were visualized using an Olympus microscope (Olympus Corporation, Japan).

### Statistical analysis

All data were generated from at least three independent experiments and analyzed using GraphPad Prism 8.0.1. (GraphPad, La Jolla, CA, USA). Continuous variables with normal distributions are expressed as mean ± standard deviation and were compared using one-way analysis of variance with post hoc Bonferroni correction. Statistical significance was defined as * #*P* < 0.05, ** ## *P* < 0.01, relative to corresponding controls.

## Results

### D-arabinose inhibits breast cancer cell growth in vitro

**Fig. 1:**
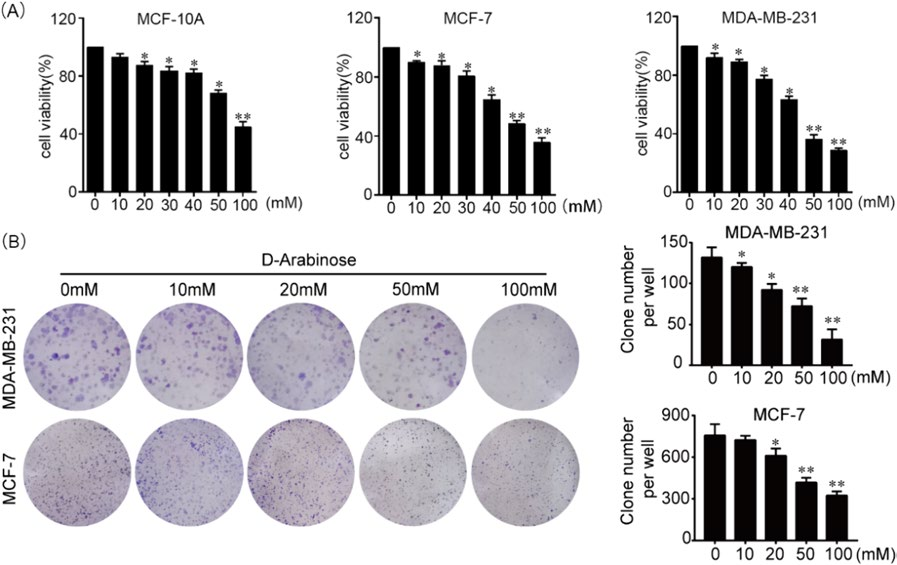
D-arabinose suppresses breast cancer cell proliferation and colony formation in vitro. **A** MCF10A, MCF-7, and MDA-MB-231cells were seeded onto 96-well plates at 1.0 × 10^3^ per well, treated with different concentrations of D-arabinose, after 72 h cell viability measured by CCK8 assay. **B** MCF-7 and MDA-MB-231 cells were seeded in 6-well plates at 1.0 × 10^3^ cells per well and colony numbers counted under a dissection microscope after D-arabinose treatment for 12 days. Results are mean values ± SEM of three independent experiments; *P < 0.05, **P < 0.01

Cancer cell growth can be restricted by treatment with natural products [12, 13]. To evaluate the role of D-arabinose in regulating the growth of breast cancer cells or normal breast cells growth, MCF-10A, MCF-7 and MDA-MB-231 cell lines were grown in complete culture medium containing different concentrations of D-arabinose. MCF-7 and MDA-MB-231 cell proliferation was significantly and dose-dependently suppressed by D-arabinose treatment, however, MCF-10A exhibited more tolerance than MCF-7 and MDA-MB-231 in D-arabinose condition (Fig. 1A). In the cell line MCF-10A, cell viability varied with the addition of D-arabinose at different concentrations, with the percentage of cell viability at 10 mM being approximately 89%, at 20 mM being approximately 86% at 30 mM being approximately 85%, at 40 mM being approximately 81%, at 50 mM being was approximately 71%, and at 100 mM, the percentage of cell viability was approximately 49%. In the cell line MCF-7, cell viability varied with different concentrations of D-arabinose added, with the percentage of cell viability being approximately 91% at 10 mM, 89% at 20 mM, 84% at 30 mM, 65% at 40 mM, 71% at 100 mM and 49% at 100 mM. 49%, and at 100 mM, the percentage of cell viability was about 38%. In the cell line MDA-MB-231, cell viability varied with the addition of different concentrations of D-arabinose, with the percentage of cell viability being approximately 95% at 10 mM, 91% at 20 mM,80% at 30 mM, 66% at 40 mM, and 50 mM. viability percentage was about 40%, and at 100 mM, the cell viability percentage was about 30%. In addition, the suppressive efficacy of D-arabinose on breast cancer cells was further confirmed by the formation of adhesive cell colonies in treated cells (Fig. 1B). These results indicate that D-arabinose can restrict breast cancer cell proliferation in vitro.

### D-arabinose arrests the breast cancer cell cycle in the G2/M phase

**Fig. 2:**
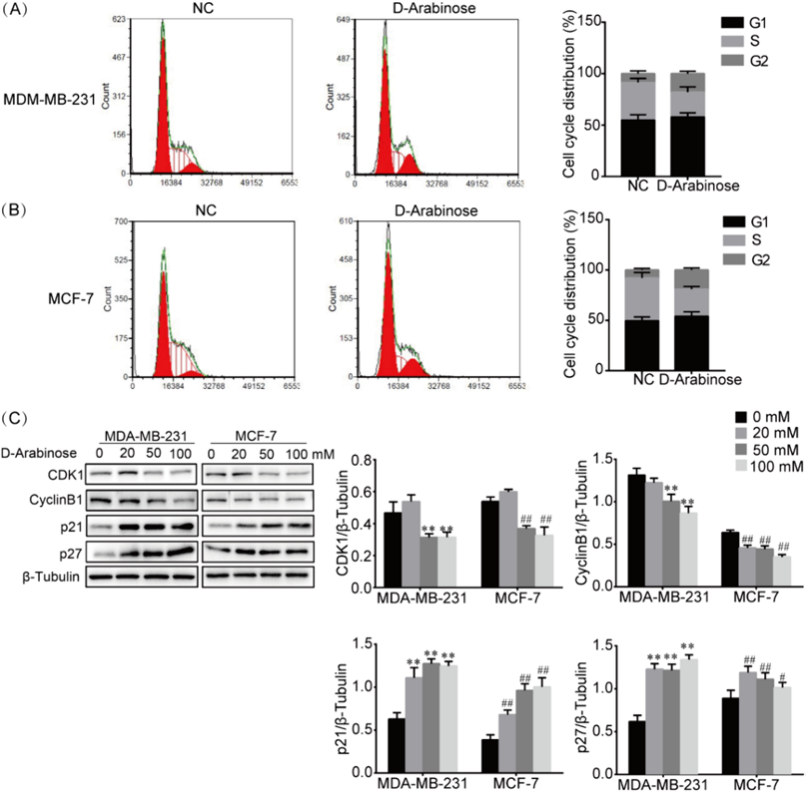
D-arabinose induces breast cancer cell cycle arrest. **A**, **B** Breast cancer cells (MCF-7 and MDA-MB-231) were treated with D-arabinose (50 mM) for 24 h. Control and treated cells were harvested, stained with 0.05% propidium iodide, and subjected to flow cytometric analysis. **C** MCF-7 and MDA-MB-231 cells were stimulated with different doses of D-arabinose (0, 20, 50, and 100 mM) for 24 h, and the expression of CDK1, Cyclin B1, p21, and p27 detected by western blot. Representative data from three separate experiments are shown. * ^#^*P* < 0.05, ** ^##^*P* < 0.01

To investigate the anti-cancer activity of D-arabinose, we assessed changes in the distribution of the breast cancer cell cycle and the expression of critical checkpoint regulators. Following D-arabinose stimulation, flow cytometry analysis showed that MCF-7 and MDA-MB-231 cells accumulated in the G2/M phase, with no influence on the number of cells in G1 phase (Fig. 2A and B). Further, after treatment of MCF-7 and MDA-MB-231 cells with D-arabinose at 20, 50, and 100 mM, the expression of CDK1, Cyclin B1, p21, and p27 were evaluated by western blot, revealing reduced expression of CDK1 and Cyclin B1 in D-arabinose-treated MCF-7 and MDA-MB-231 cells, while levels of p21 and p27 were elevated (Fig. 2C). These data indicate that D-arabinose inhibits BC cell growth by blocking cell cycle progression at the G2/M phase.

### D-arabinose induces autophagy in breast cancer cells

**Fig. 3:**
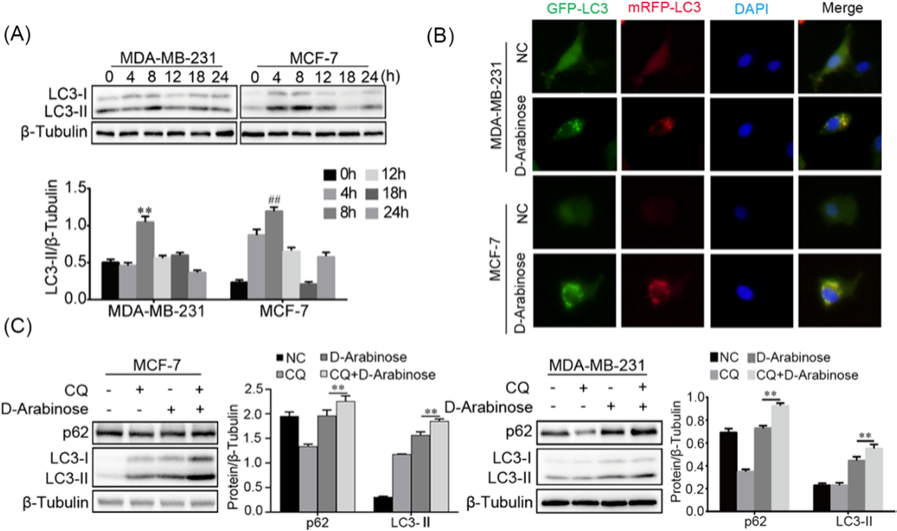
Autophagy was induced by D-arabinose in breast cancer cells. **A** MDA-MB-231 and MCF-7 were stimulated with D-arabinose (50 mM) and LC3-II expression detected by western-blot at different time points. **B** After infection with mRFP-GFP-LC3 overexpression adenovirus, MCF-7 and MDA-MB-231 cells on slides were treated with D-arabinose (50 mM) for 8 h, then analyzed by confocal microscopy. LC3-II puncta-positive cells in five images from each group were counted. **C** MCF-7 and MDA-MB-231 cells were treated with or without chloroquine (CQ) before exposure to D-arabinose (50 mM). Expression levels of p62 and LC3-II were analyzed by western blot. Representative data from three separate experiments are shown. AV, autophagic vacuoles. * ^#^*P* < 0.05, ** ^##^*P* < 0.01

In cancer cells, cell cycle phase of is closely regulated by autophagy in different contexts [14, 15]. To assess whether autophagy is triggered by D-arabinose, autophagy activation of breast cancer cells cultured with D-arabinose was assessed at different time points. D-arabinose treatment resulted in prominent elevation of LC3-II in MDA-MB-231 and MCF-7 cells with the most significant augmentation observed at 8 h (Fig. 3A). Moreover, mRFP-GFP-LC3 adenovirus was applied and the formation of autophagosomes observed by transmission electron microscopy. The results demonstrated that D-arabinose increased the number of cytoplasmic autophagosomes (Fig. 3B). To further evaluate autophagic flux during D-arabinose treatment, the autophagy inhibitor, chloroquine (CQ), was used to arrest autophagy flux in MDA-MB-231 cells. After treatment with CQ, p62 and LC3-II induction was further enhanced in MDA-MB-231 cells (Fig. 3C). These findings suggest that exposure of breast cancers to D-arabinose can indeed mediate autophagy.

### D-arabinose regulated cell cycle arrest via the autophagic pathway

**Fig. 4:**
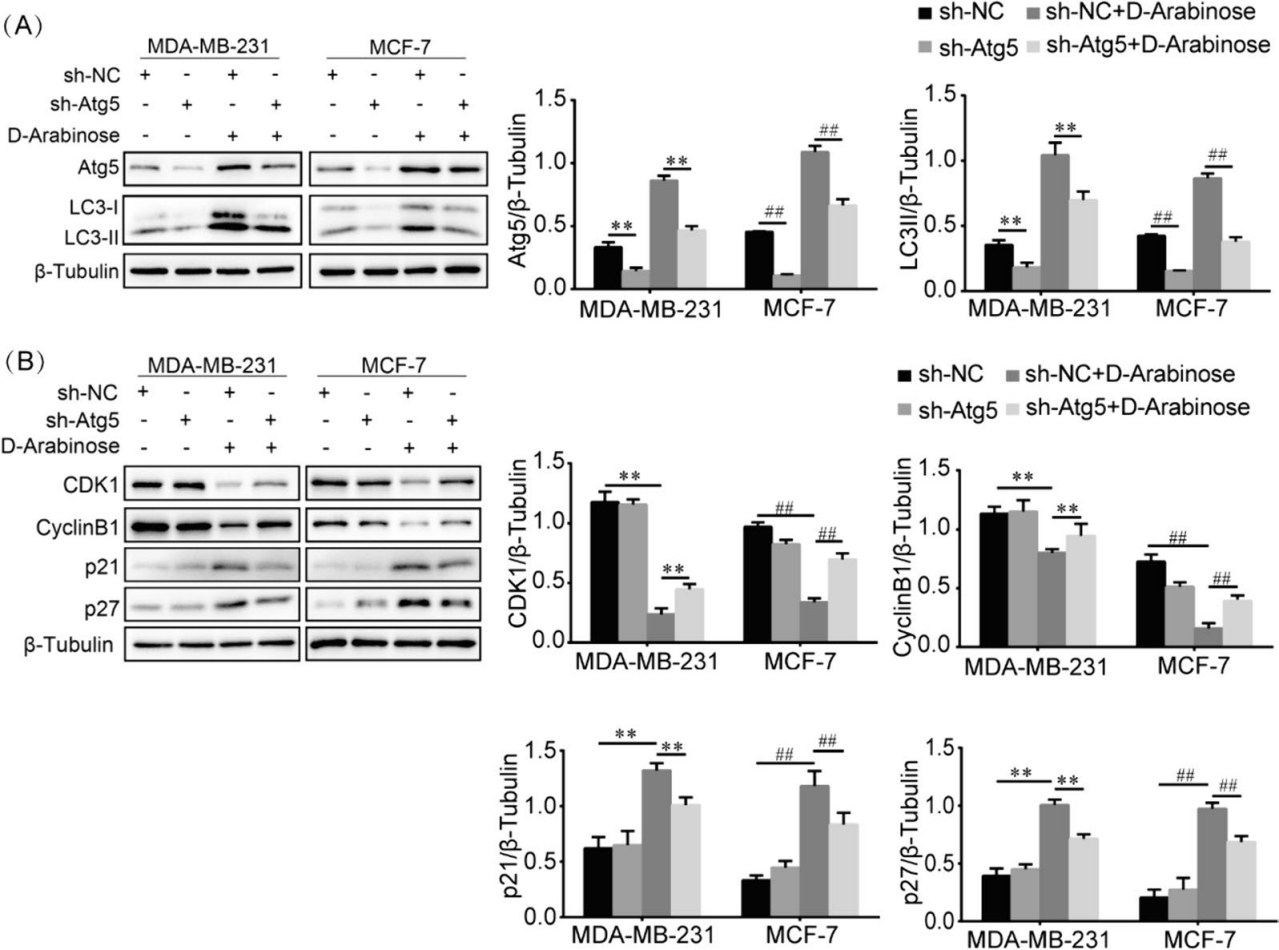
Effects of autophagy induced by D-arabinose on cell cycle arrest in breast cancer cells. **A** MCF-7 and MDA-MB-231 cells were infected with control lentivirus (sh-NC) or lentivirus-expressing shRNA targeting Atg5 (sh-Atg5), and treated with or without D-arabinose (50 mM). Expression of Atg5 and LC3-II was analyzed by western blot. **B** Western blot analysis to measure the protein expression levels of CDK1, Cyclin B1, p21, and p27 in sh-Atg5 lentivirus-infected MDA-MB-231 and MCF-7 cells after treatment with D-arabinose. Representative data from three separate experiments are shown. * ^#^*P* < 0.05, ** ^##^*P* < 0.01

To determine whether the effect of D-arabinose on cell cycle arrest was triggered by autophagy, lentivirus expressing shRNA specific for Atg5 (sh-Atg5) was used to inhibit MDA-MB-231 and MCF-7 cells autophagy, and the expression proteins associated with the cell cycle measured in presence or absence of D-arabinose. The control group comprised MDA-MB-231 and MCF-7 cells infected with lentivirus expressing scrambled shRNA (sh-NC). Atg5 knockdown significantly inhibited autophagy induced by exposure to D-arabinose, as illustrated by decreased Atg5 and LC3-II expression compared with sh-NC-treated controls (Fig. 4A). Further, the results indicate that Atg5 knockdown in MDA-MB-231 and MCF-7 cells treated with D-arabinose remarkably increased the expression of CDK1 and Cyclin B1, while it suppressed the expression of p21 and p27 compared with sh-NC treated cells (Fig. 4B). These findings provide solid evidence that cell cycle arrest in response to D-arabinose treatment is mediated by autophagy of breast cancer cells, while inhibition of autophagy counteracted the suppressive effect of D-arabinose.

### Inhibition of autophagy interrupts cell cycle arrest by p38 MAPK pathway

**Fig. 5:**
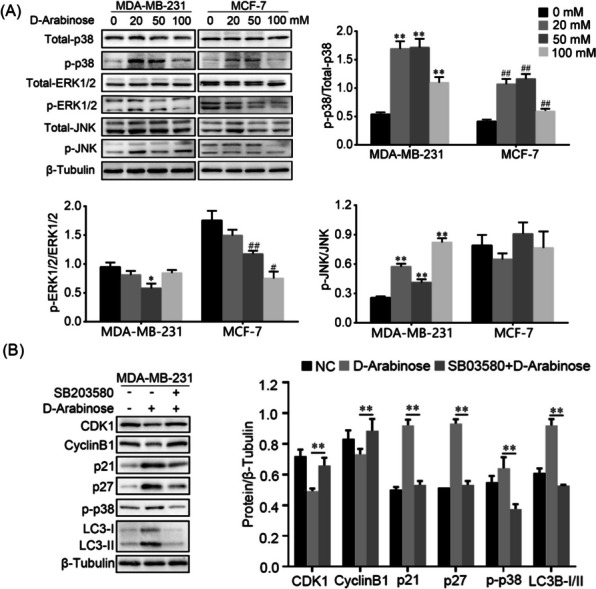
D-arabinose induces cell cycle arrest via autophagy-dependent p38 MAPK signaling in breast cancer cells. **A** Breast cancer cells (MCF-7 and MDA-MB-231) were conditioned with or without various concentration of D-arabinose (0, 20, 50, and 100 mM), total cell proteins harvested, and expression levels of JNK, ERK1/2, p38, and their phosphorylated forms measured by western blot. **B** MDA-MB-231 cells were treated with the p38 MAPK inhibitor, SB203580 (10 μM), for 2 h prior to D-arabinose (50 mM) treatment, and expression levels of LC3-II, CDK1, Cyclin B1, p21, and p27 assessed by western blot. Representative data from three separate experiments are shown. * ^#^*P* < 0.05, ** ^##^*P* < 0.01

Next, we investigated the mechanisms underlying cell cycle arrest via autophagy following D-arabinose treatment of breast cancer cells. The MAPK signaling pathway contributes to regulation of autophagy induced by various stress conditions, resulting in modulation of cellular functions [16, 17]. As expected, we observed that levels of phosphorylated p38 (p-p38) were significantly increased on exposure of both MDA-MB-231 and MCF-7 cells to different concentrations of D-arabinose; however, the trend in changes to phosphorylated JUN (p-JUN) and ERK1/2 (p-ERK1/2) expression was not consistent between MDA-MB-231 and MCF-7 cells (Fig. 5A). Furthermore, the p38 MAPK inhibitor, SB203580, suppressed autophagy mediated by D-arabinose by down-regulating LC3-II expression, which enhanced the expression levels of CDK1 and Cyclin B1, but reduced those of p21 and p27 (Fig. 5B). Together, these results demonstrated that D-arabinose induced autophagy lead to cell cycle arrest through activation of the p38 MAPK signaling pathway in breast cancer cells.

### D-arabinose represses breast tumor growth in vivo

**Fig. 6:**
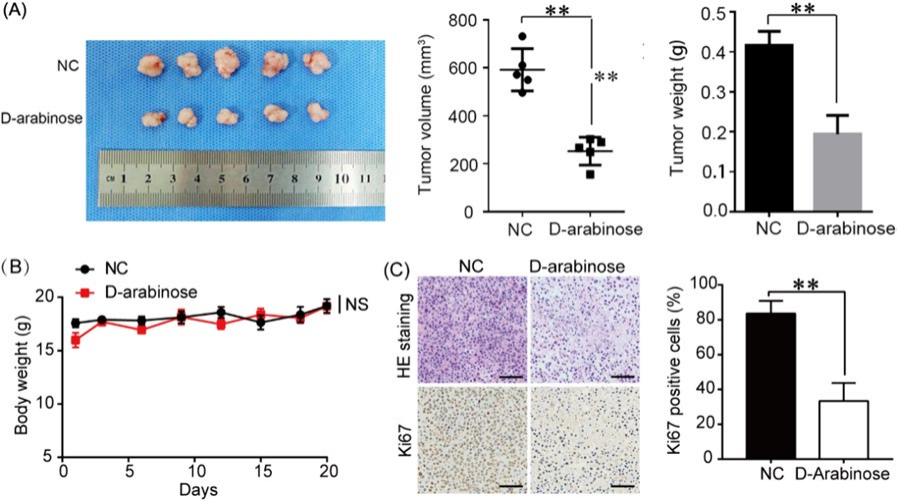
D-arabinose remarkably decreases the growth of breast cancer xenograft tumors. Nude mice were inoculated with 2 × 10^6^ human MDA-MB-231 cells each. One week after cell injection, mice in the treatment group were administered 2.5 g/kg D-arabinose by gavage every day for 4 weeks; control mice received the same volume of solvent. **A** Images of tumor masses from each group. Changes in tumor volume and weight after D-arabinose treatment were quantified in each group. **B** Nude mice body weight during D-arabinose treatment. **C** Tumor tissue sections stained with hematoxylin–eosin and for Ki67 by immunohistochemistry. All data are presented as mean ± SD. *P < 0.05, **P < 0.01, NS not significant

In order to explore the function of D-arabinose in tumor formation, a tumor xenograft model was generated by subcutaneous injection of MDA-MB-231 cells into the fat pads of female BALB/c nude mice. One week after cell injection, tumors were treated with D-arabinose (2.5 g/kg/day) via gavage for a further 20 days. The results showed that D-arabinose reduced tumor size (Fig. 6A), without prominently influencing mouse weight (Fig. 6B). Hematoxylin–eosin staining revealed that tumors had a loose structure, with increased inflammatory cell infiltration in tumor tissues treated with D-arabinose (Fig. 6C). Further, Ki-67 expression was lower in tumors from D-arabinose treated mice than in those from controls (Fig. 6C). Together, these results suggested that D-arabinose could suppress breast cancer progression by inducing cell cycle arrest in vivo.

## Discussion

Treatment of breast cancer remains challenging, due to poor responses, drug resistance, and intolerance in a significant proportion of patients. Hence, there is an urgent need to explore new drugs or therapeutic targets to achieve improvements in clinical efficacy [18–20]. Recently, monosaccharides derived from natural products have been the subject of increasing attention for use in prevention and treatment for various types of cancer [21]. D-arabinose is unlike other monosaccharides, as humans cannot derive it naturally from food. Although D-arabinose contributes to the regulation of various biological activities, such as metabolism and cell proliferation, there was previously no direct evidence suggesting that D-arabinose has anti-cancer effects by inhibiting tumor cell growth [22, 23]. In present study, we found that D-arabinose could significantly restrict the proliferation of breast cancer cells in vivo* and *in vitro. Similar inhibitory efficacy of D-arabinose has previously been observed in another eukaryote, Caenorhabditis elegans [8]. More importantly, normal breast cells present more resistance than cancer cells under D-arabinose stimulation, imply that D-arabinose is adapt to clinical application. Collectively, these results represent the first demonstration that D-arabinose has significant anti-cancer activity against breast cancer cells. In clinical applications, D-arabinose is expected to exhibit efficacy and safety, even in combination with chemotherapeutic drugs, due to its compatibility with human physiology.

In addition to the role of D-arabinose in cancer, the role of monosaccharides in cancer is also a hot topic at this stage. Firstly, glucose plays an important role in cancer metabolism. In order to meet their rapidly growing needs, cancer cells usually increase the uptake and utilization of glucose to obtain more energy, a phenomenon known as the “Warburg effect”. Glucose not only provides energy for cancer cells, but also participates in the survival, proliferation, invasion and metastasis of cancer cells. Therefore, interventions targeting the glucose metabolism of cancer cells have become an important research direction in cancer therapy [24–26]. Secondly, galactose also plays an important role in the development of cancer. Galactose affects cell recognition and signaling by participating in the synthesis of cell surface glycoproteins and glycolipids. It has been found that the galactose modification pattern on the surface of certain cancer cells has changed, and this change is closely related to the invasiveness and metastatic ability of cancer cells. For example, abnormalities in galactose modification may promote tumor cells to evade immune surveillance, thus contributing to cancer progression. Therefore, by studying the modification patterns of galactose on the surface of cancer cells, new strategies for cancer diagnosis and treatment could be provided [27].Finally, N-acetylneuraminic acid (sialic acid) is a special monosaccharide that occupies an important position in the modification of glycan chains on the surface of cancer cells.The content and distribution of N-acetylneuraminic acid tend to be different in cancer cells from that of normal cells, and this difference has been associated with the characteristics of cancer cells such as invasiveness, metastatic ability and immune escape. Therefore, N-acetylneuraminic acid and its related signaling pathways have become one of the hot spots in cancer research [28, 29].

Aberrant cell cycle progression is an important feature in limiting the proliferation of tumor cells, and therapy targeting cell cycle progression is considered a promising anti-cancer strategy [30, 31]. The regulatory relationships among cyclins, cyclin-dependent kinases (CDKs), CDK inhibitors (CKIs), and the eukaryotic cell division cycle are established [32]. The Cdc2-cyclin B complex, also known as M-phase promoting factor, is responsible for cell cycle phase transition and mitotic entry, and inhibition of its activity arrests the cell cycle at G2 phase [33]. p21 and p27 belong to the Cip/Kip family of cyclin-dependent kinase CKIs, which inhibit the CDK-cyclin activity and restrain cell cycle progression [34]. In this study, flow cytometry analysis indicated that D-arabinose arrests breast cancer cells at G2/M phase. Further, we assessed the expression of proteins associated with G2/M phase, and confirmed that D-arabinose can elevate the expression of p21 and p27, and down-regulated that of CDK1 and Cyclin B1. High expression of CDK1 and Cyclin B1 and low levels of p21/p27 are frequently detected in various types of cancer and associated with cancer development and poor prognosis [35–37]. Together, these results illustrate that D-arabinose has a pivotal role in modulation of cell cycle progression by regulating the activity of CDK1, Cyclin B1, p21, and p27 in breast cancer cells.

Autophagy is a ubiquitous, highly conserved, self-protective mechanism through which damaged organelles, misfolded proteins, and invading pathogens are sequestered within vesicles and delivered to lysosomes for degradation or recycling, thus maintaining intracellular environmental homeostatic stability in eukaryotic cells [38]. The role of autophagy in D-arabinose regulated cell cycle arrest has not been reported previously; therefore, this is the first study to assess whether autophagy can be attributed to cell cycle arrest mediated by D-arabinose in breast cancer cells. LC3-II, derived from cleaved LC3-I, is a pivotal element of autophagy, which represents the progress of autophagy and the number of autophagosomes, in certain contexts [39]. In this study, our results indicate that levels of LC3-II increased in a time-dependent manner following D-arabinose exposure. Further, autophagosome formation was confirmed, based on the number of puncta in the cytoplasm. Furthermore, we assessed autophagy flux using autophagy inhibitors. As a late autophagy inhibitor CQ blocks the fusion of autophagosomes and lysosomes, leading to accumulation of large numbers of autophagosomes and augmented levels of LC3-II and p62 [40]. Our data show that CQ treatment dramatically increased LC3-II and p62 levels induced by D-arabinose compared with the control group. These results clarify that D-arabinose can induce breast cancer cell autophagy. In addition, there is ample evidence demonstrating that various cell cycle regulators contribute to modulation of autophagic processes. For example, inhibition of CDK1 promotes the recovery of motor neuron function after spinal cord injury by suppressing the activation of neuron apoptosis and autophagy [41]. Further, constitutive expression and accumulation of p21 and p27 arrest the cell cycle at G0/S phase by inducing autophagy in non-small cell lung cancer and head and neck squamous cancer cells [42, 43]. The results of the present study show that autophagy initiated by D-arabinose is accompanied by down-regulation of CDK1 and Cyclin B1 and up-regulation of p21 and p27, suggesting that D-arabinose may modulate autophagy via cell cycle regulators in breast cancer cells. Intriguingly, a previous study clarified that autophagy plays a pivotal role in regulating cell cycle arrest of proximal epithelial cells, and that enhanced expression of Atg5 could suppress renal fibrosis by rescuing G2/M arrest [44]. In contrast, Qiang Ma et al. found that autophagy deficiency induced by dihydroartemisinin blocked cell cycle arrest at the G2/M phase in esophageal cancer cells [15]. Nevertheless, whether or not autophagy contributes to cell cycle progression of breast cancer cells treated with D-arabinose as an upstream regulator has yet to be confirmed. In this study, inhibition of autophagy by specific downregulation of Atg5 using a lentivirus expressing a targeted shRNA, interrupted breast cancer cell cycle arrest in response to D-arabinose treatment, observed as increased expression of CDK1 and Cyclin B1, as well as reduced levels of p21 and p27 and enhanced cell proliferation potential. These results suggest that autophagy contributes to modulation of cell cycle regulators, and determines the cell cycle progression of breast cancer cells treated with D-arabinose. Collectively, our data reveal that D-arabinose can induce cell cycle arrest at the G2/M phase in an autophagy-dependent manner, indicating that enhancement of autophagy may strengthen its anti-breast cancer efficacy.

JNK, ERK1/2, and p38 MAPK are members of the MAPK signaling pathway, which plays a pivotal role in regulation of cell growth, differentiation, senescence, and stress responses under various extracellular stimuli, via modulating the activity of downstream transcription factors [45, 46]. MAPK family members are established as involved in regulation of autophagy and cell cycle arrest [47, 48]. To explore the mechanism by which D-arabinose regulates autophagy and the cell cycle, we investigated MAPK signaling pathway protein expression levels in breast cancer cells. We found that D-arabinose treatment significantly increased the expression of p-p38 MAPK in both breast cancer cell lines tested (MCF-7 and MDA-MB-231). Previous studies also confirmed that the p38 MAPK pathway can influence the cell cycle to control breast cancer cell proliferation [49, 50]. Intriguingly, p-JNK and p-ERK1/2 did not exhibit the same expression tendencies in MCF-7 and MDA-MB-231 cells. This phenomenon may be attributable to differences in molecular classification between these two cell lines: MCF-7 is derived from luminal A type breast cancer, where MDA-MB-231 is a triple-negative breast cancer cell line. Furthermore, we observed that the p38 MAPK inhibitor, SB203580, synergistically reversed the expression of autophagy and cell cycle arrest associated proteins, suggesting that p38 MAPK signaling contributes to D-arabinose-mediated autophagy and cell cycle arrest in breast cancer cells.

Our study found for the first time the role of D-arabinose in breast cancer, especially Luminal A and TNBC, but due to the different molecular typing and heterogeneity of breast cancer, we need to study the different molecular typing of breast cancer separately, which is the next step of our research team and more in-depth exploration. D-arabinose has the promise to be used as a breast cancer D-arabinose, due to its water solubility, has the potential to be introduced as an anticancer drug for breast cancer, providing a theoretical basis for further treatment of breast cancer.

## Conclusions

In summary, the results of the present study demonstrate that D-arabinose has potent anti-tumor efficacy against breast cancer, which may be attributable to induction of cell cycle arrest at G2/M phase and autophagy. Moreover, we found that the anti-tumor potential of D-arabinose is closely associated with p38 signaling pathway activation, regulating the cell cycle and autophagy processes in Luminal A and Triple Negative Breast Cancer cells. These results provide solid evidence supporting the potential of D-arabinose as a novel breast cancer treatment.

### Supplementary Information

**Supplementary Material 1.**

## Data Availability

The data that support the findings of this study are available from the corresponding author upon reasonable request.
