# Supplementary material for: Pectinose induces cell cycle arrest in luminal A and triple-negative breast cancer cells by promoting autophagy through activation of the p38 MAPK signaling pathway
Source: BMC Cancer. 2024 May 24;24:639. doi: 10.1186/s12885-024-12293-8 (PMC11127404; doi:10.1186/s12885-024-12293-8)

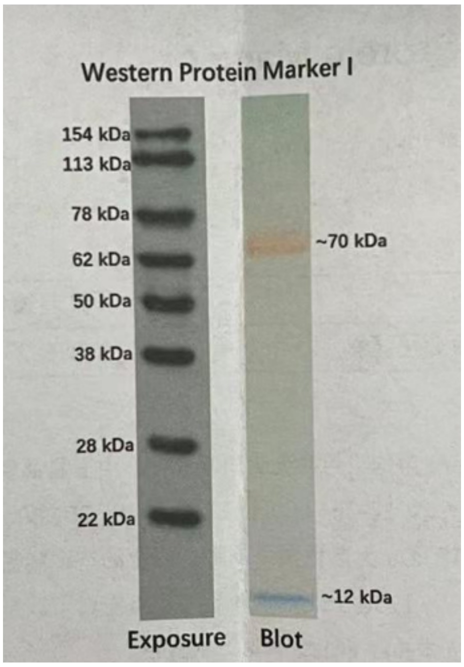


Original Image For Fig 2C
MDA-MB-231
Cyclin B1

p21

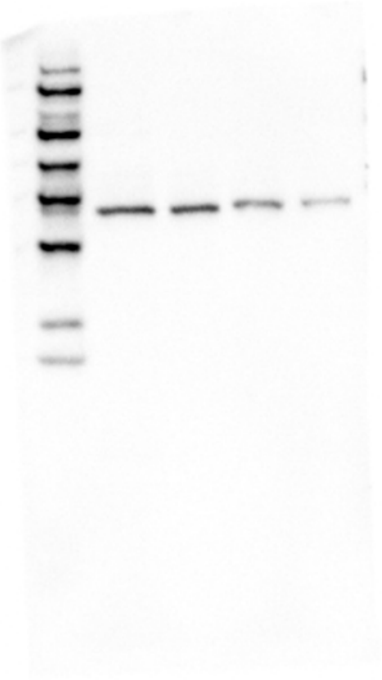

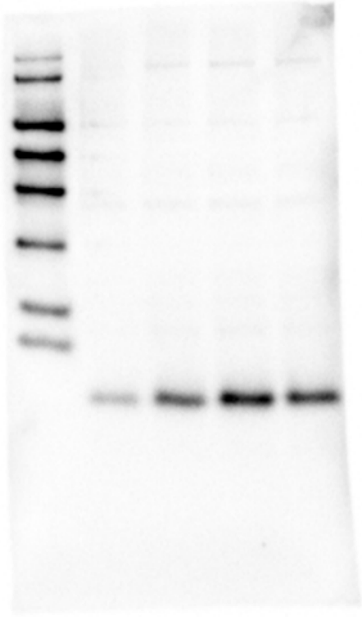

p27

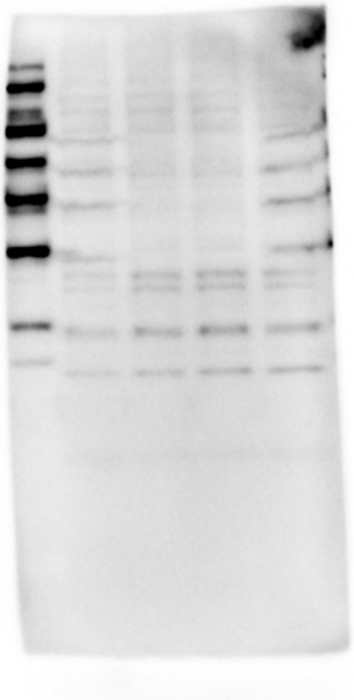

β-Tubulin

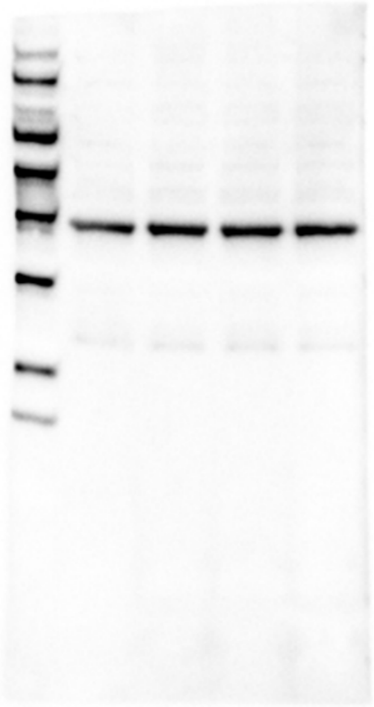

Original Image For Fig 2C MCF-7

Cyclin B1

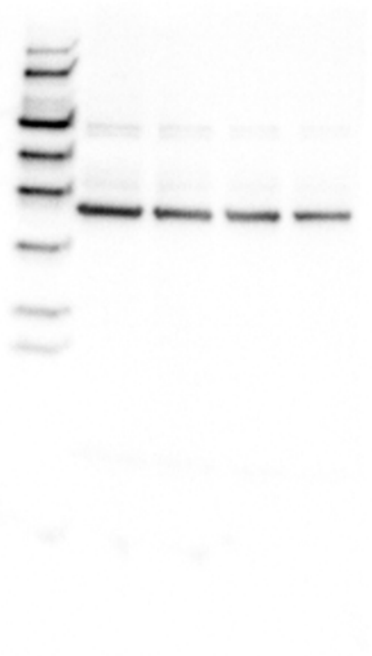

p21

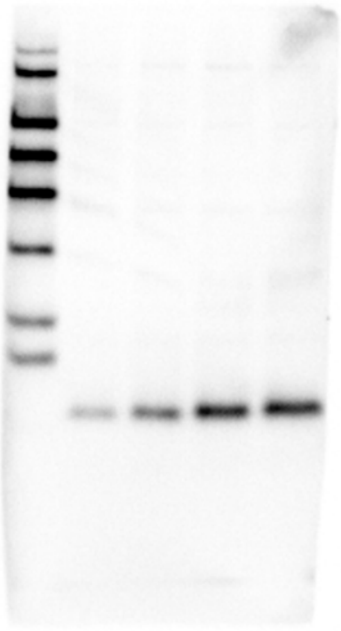

p27

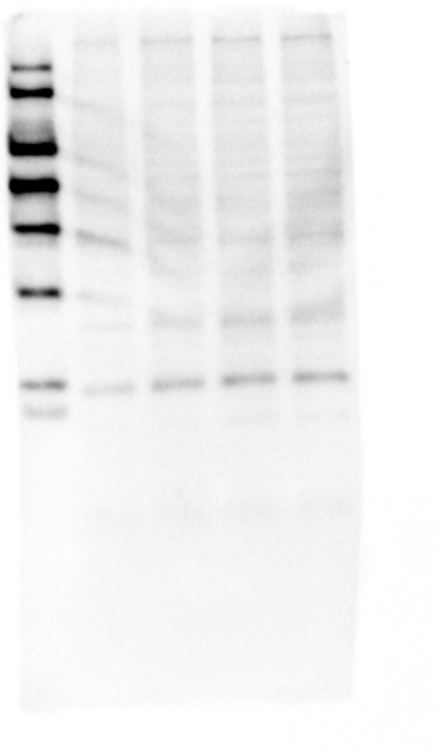

β-Tubulin

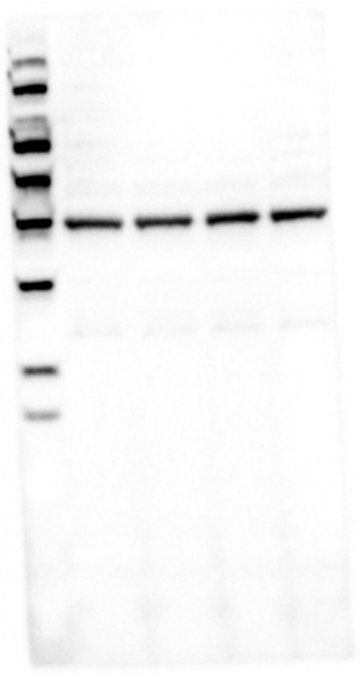

Original Image For Fig 3A MDA-MB-231
LC3

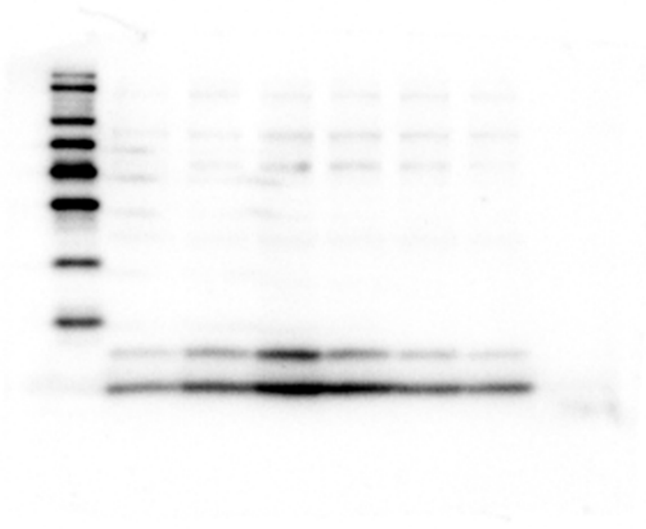

β-Tubulin

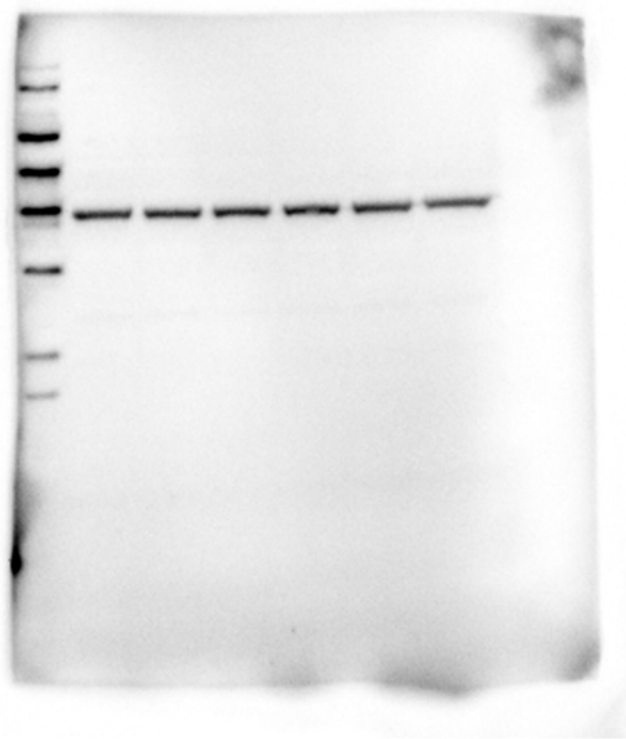

Original Image For Fig 3A MCF-7
LC3

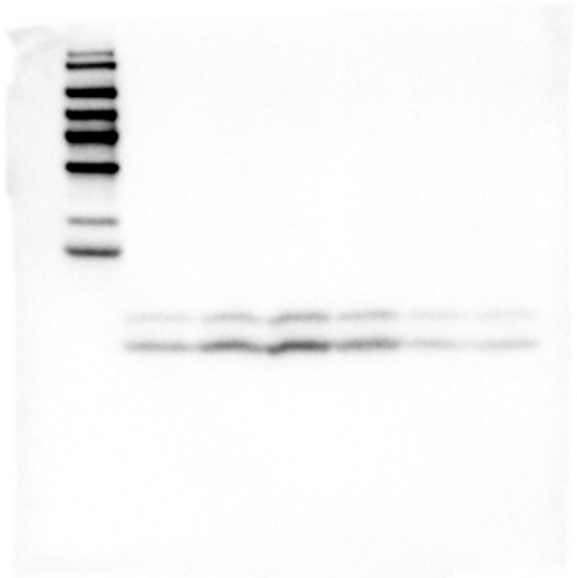

β-Tubulin

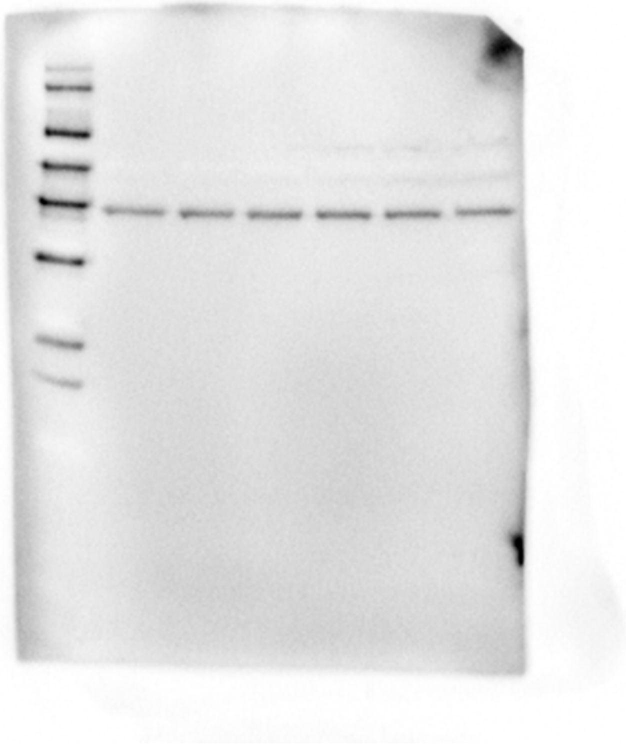

Original Image For Fig 3B
MDA-MB-231 NC GFP

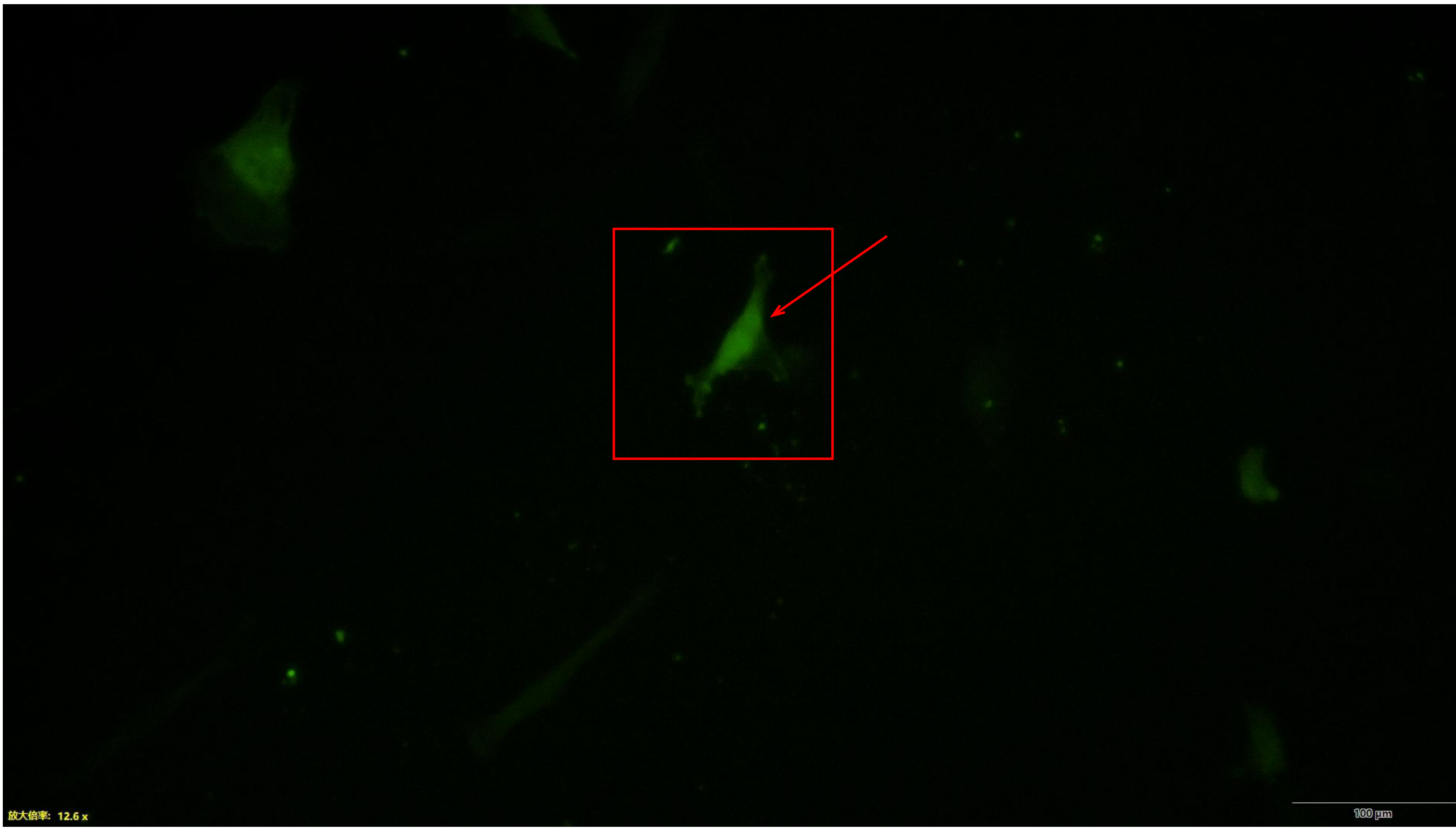


mRFP

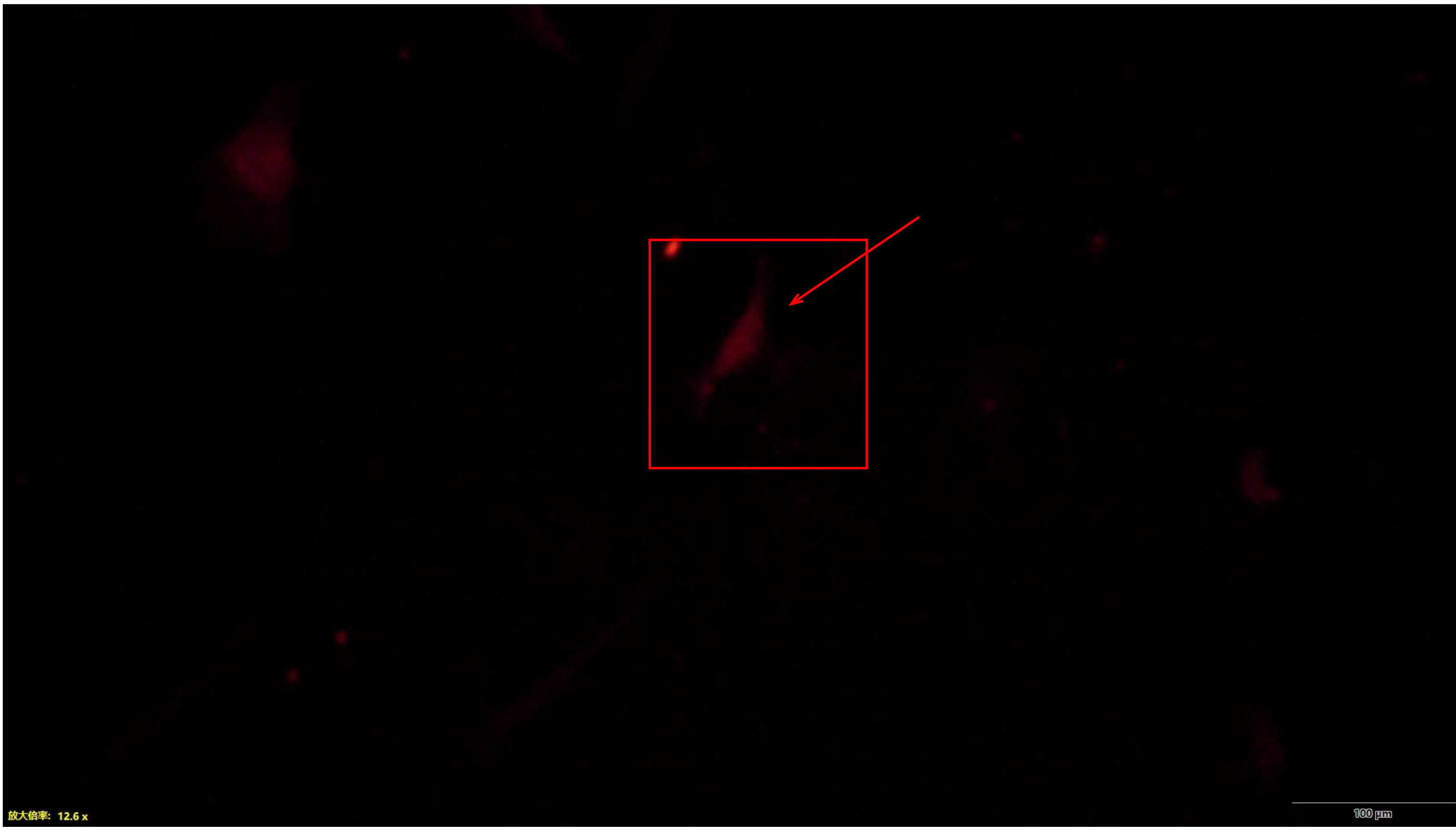

Original Image For Fig 3B
MDA-MB-231 NC DAPI

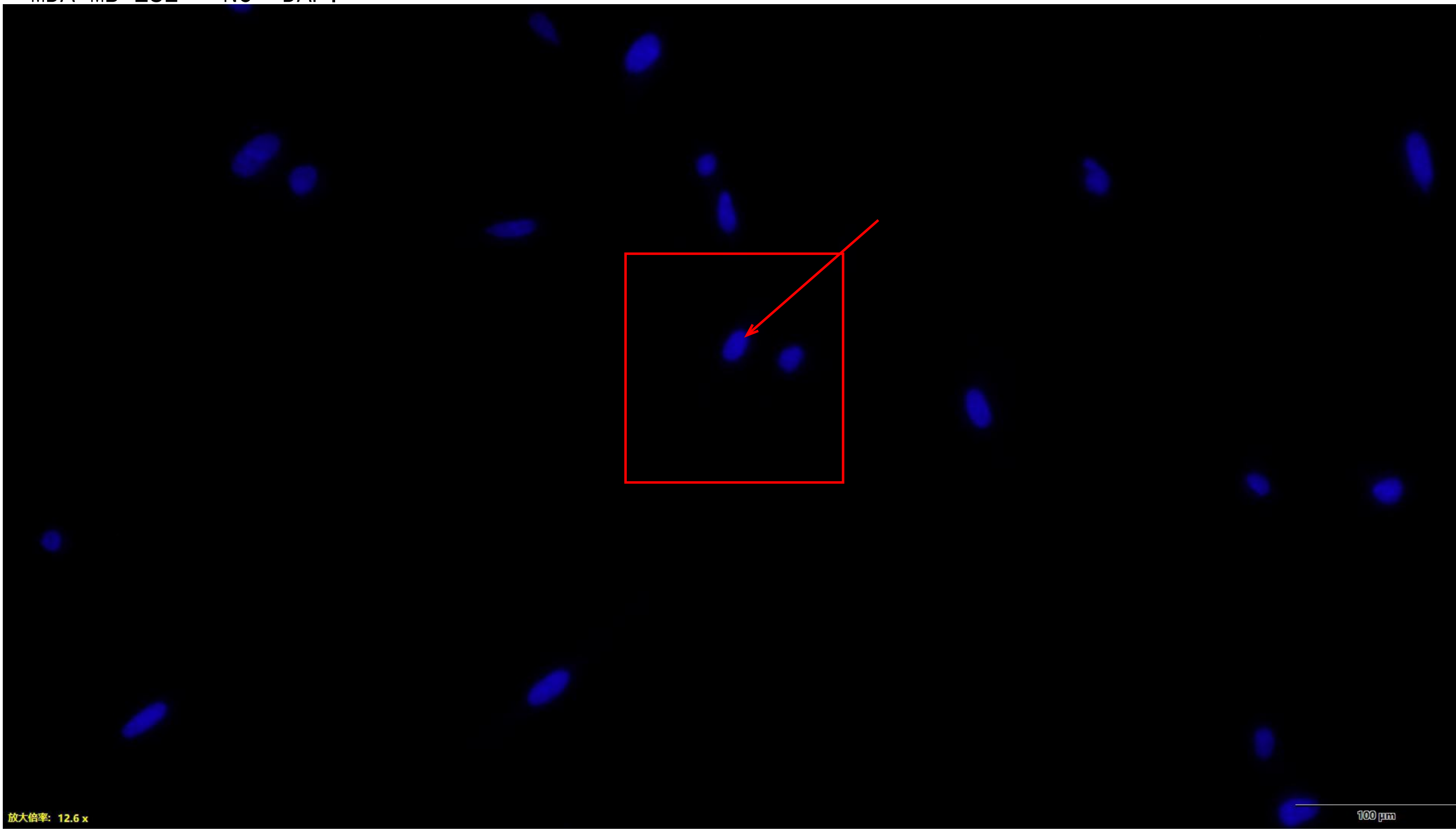


Merge

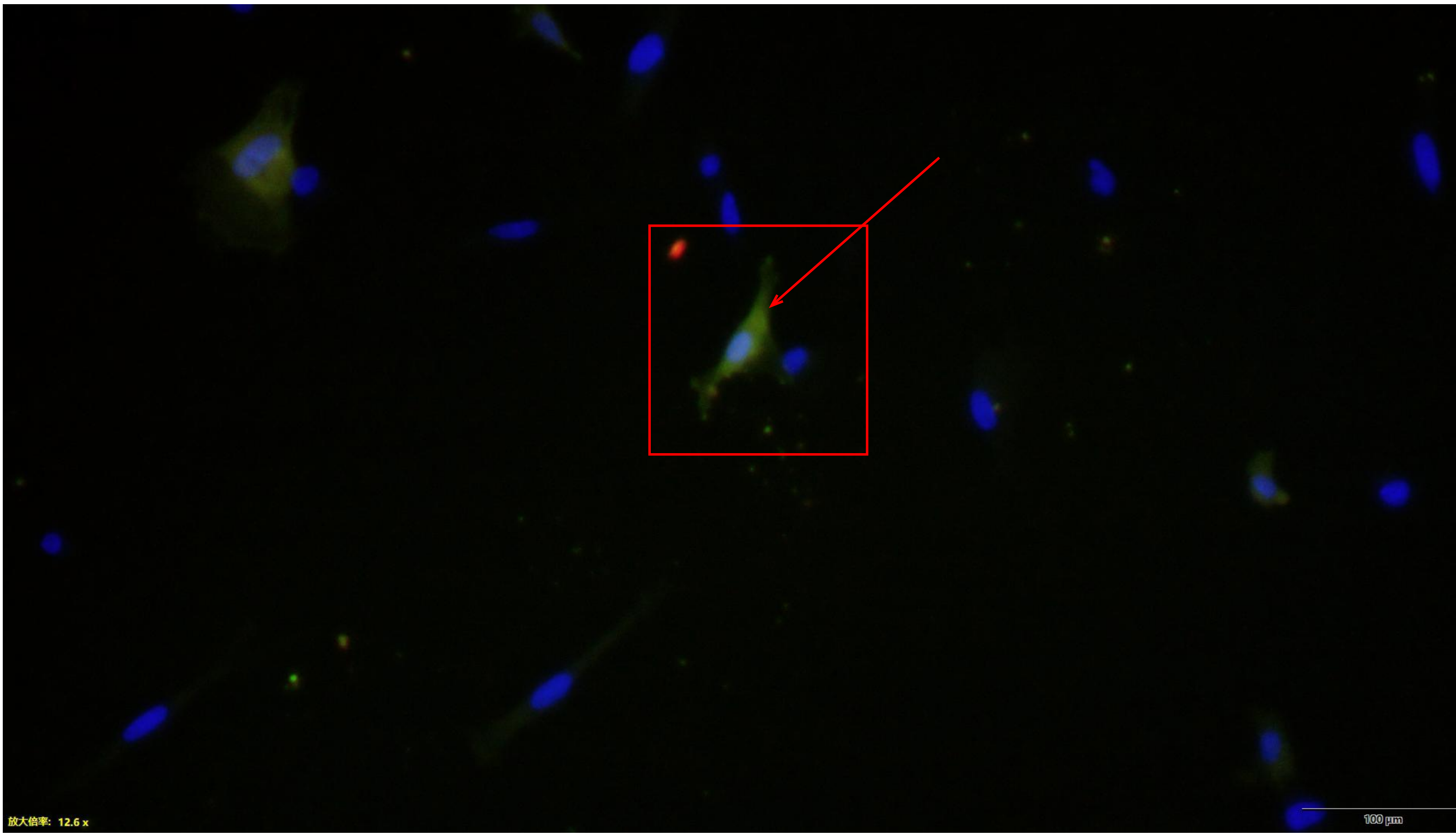

Original Image For Fig 3B MDA-MB-231 Treatmet GFP

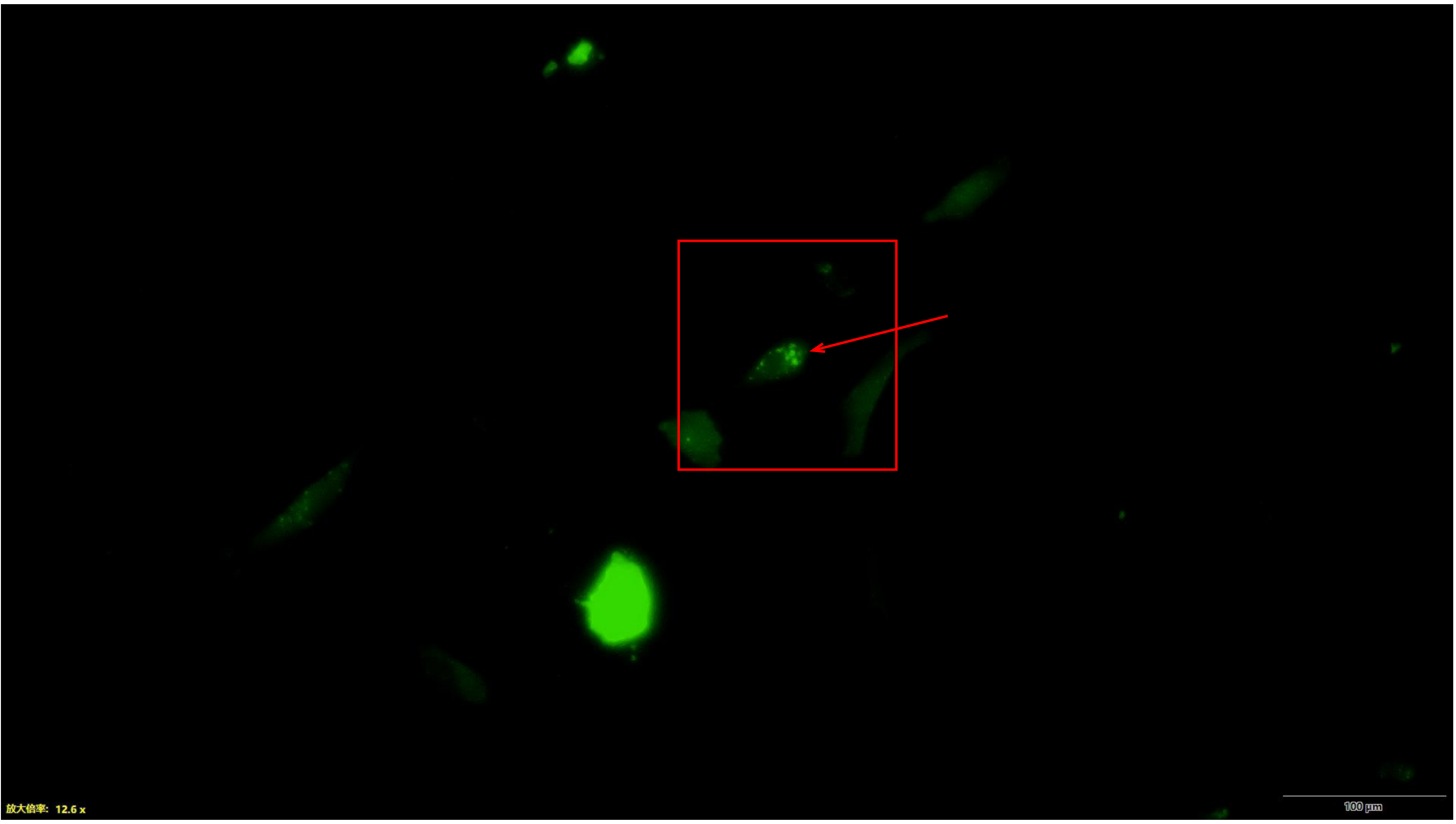


mRFP

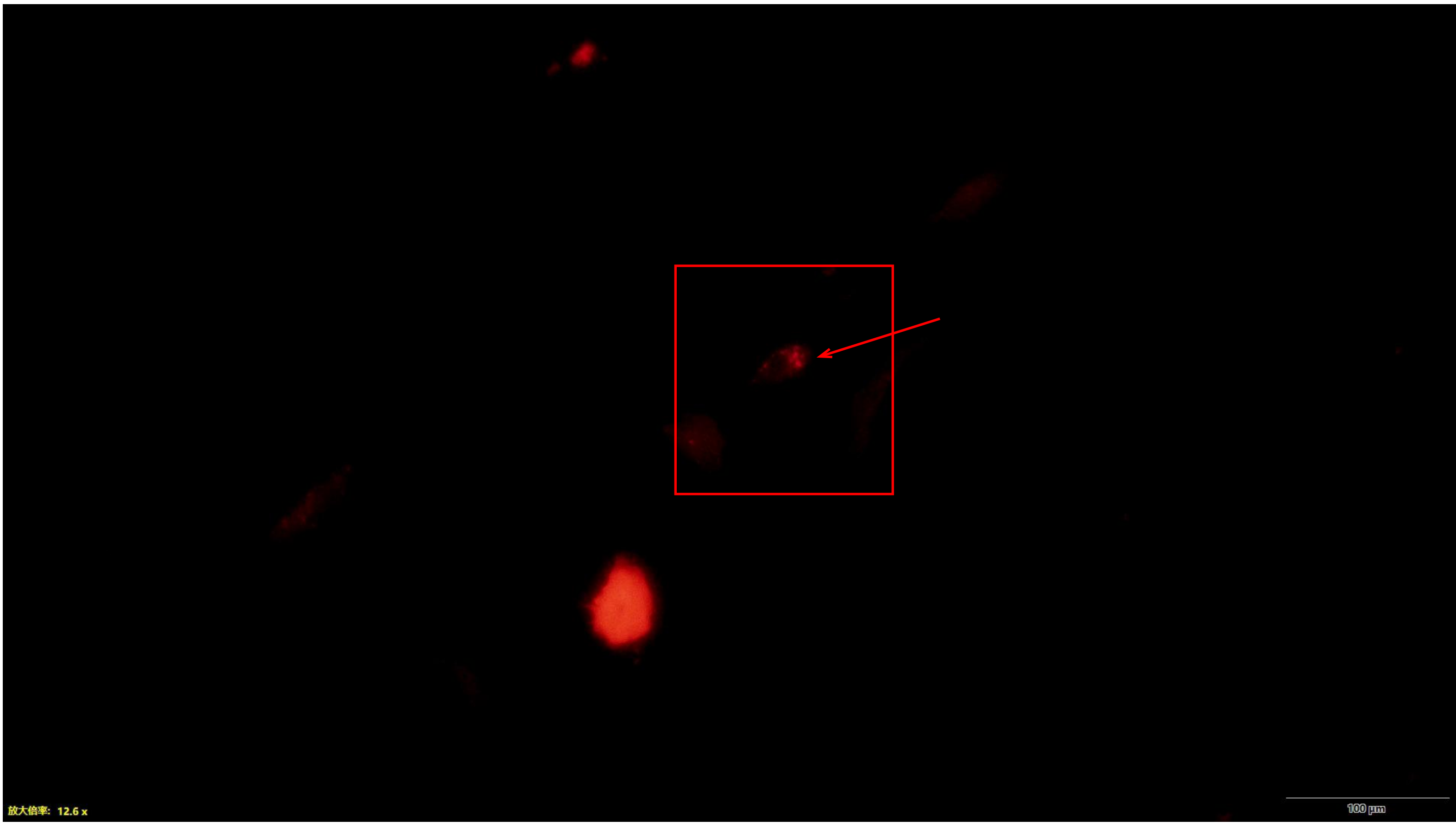

Original Image For Fig 3B MDA-MB-231

Treatment DAPI

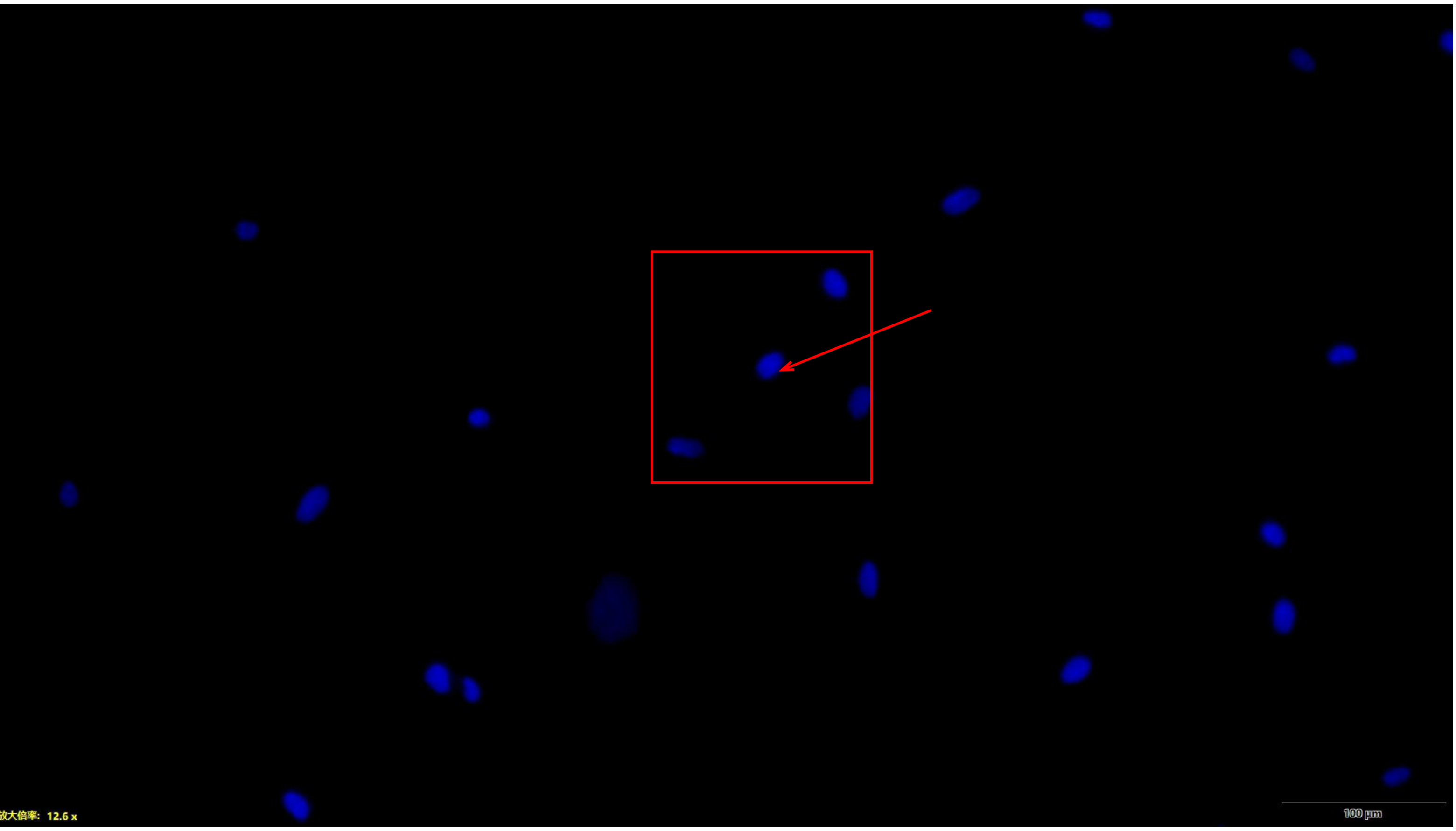


Merge

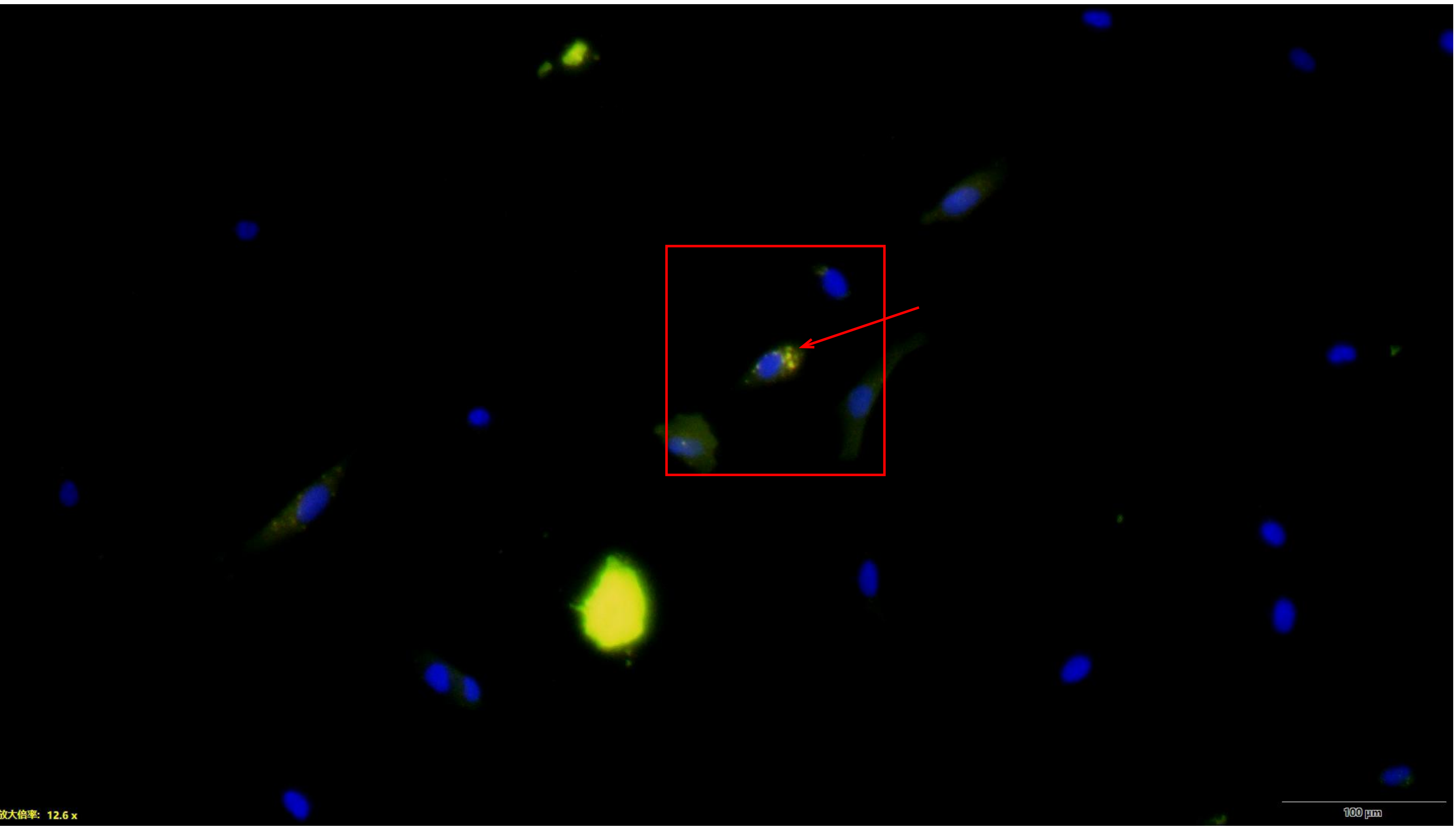

Original Image For Fig 3B
MCF-7 NC GFP

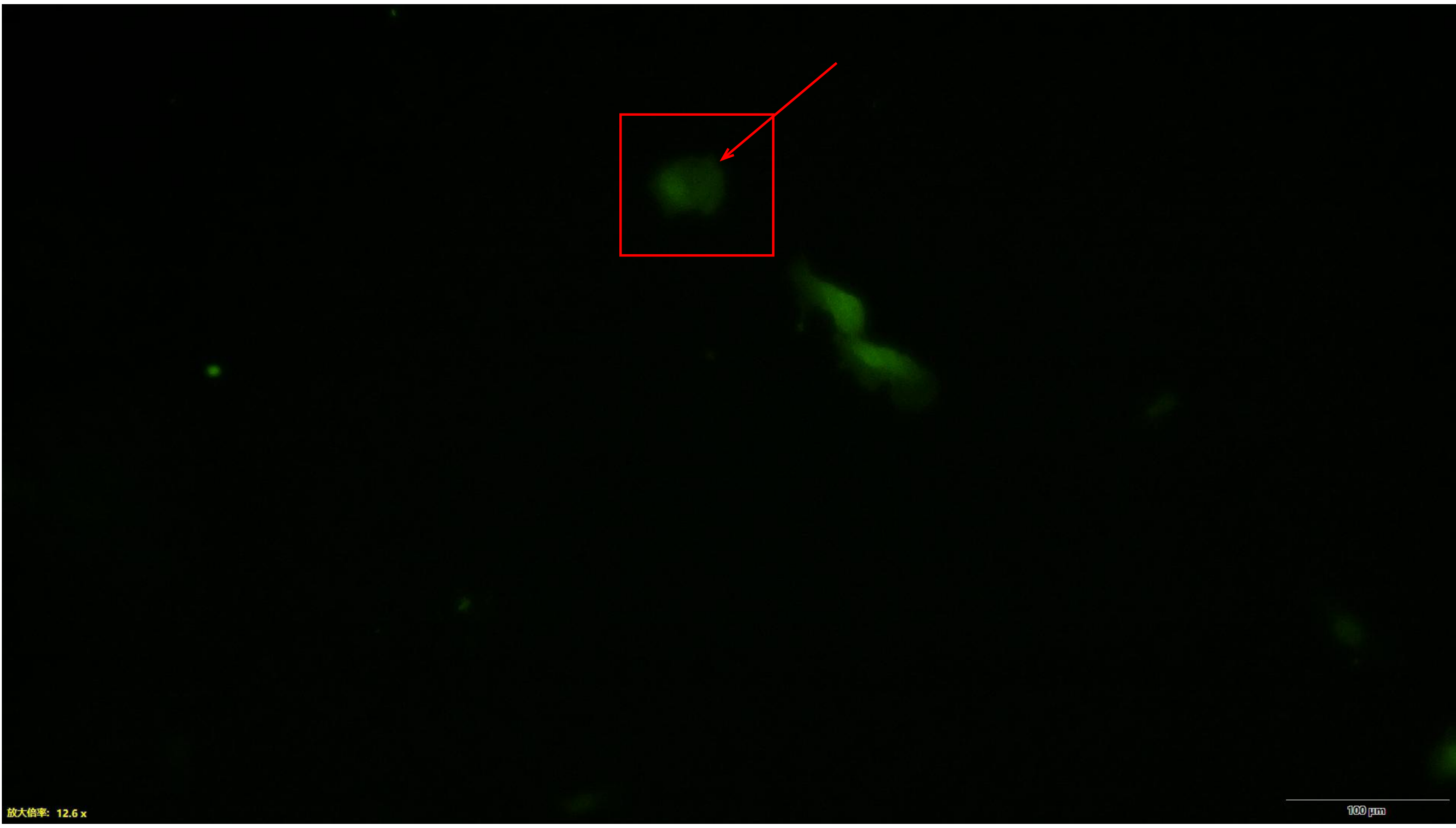


mRFP

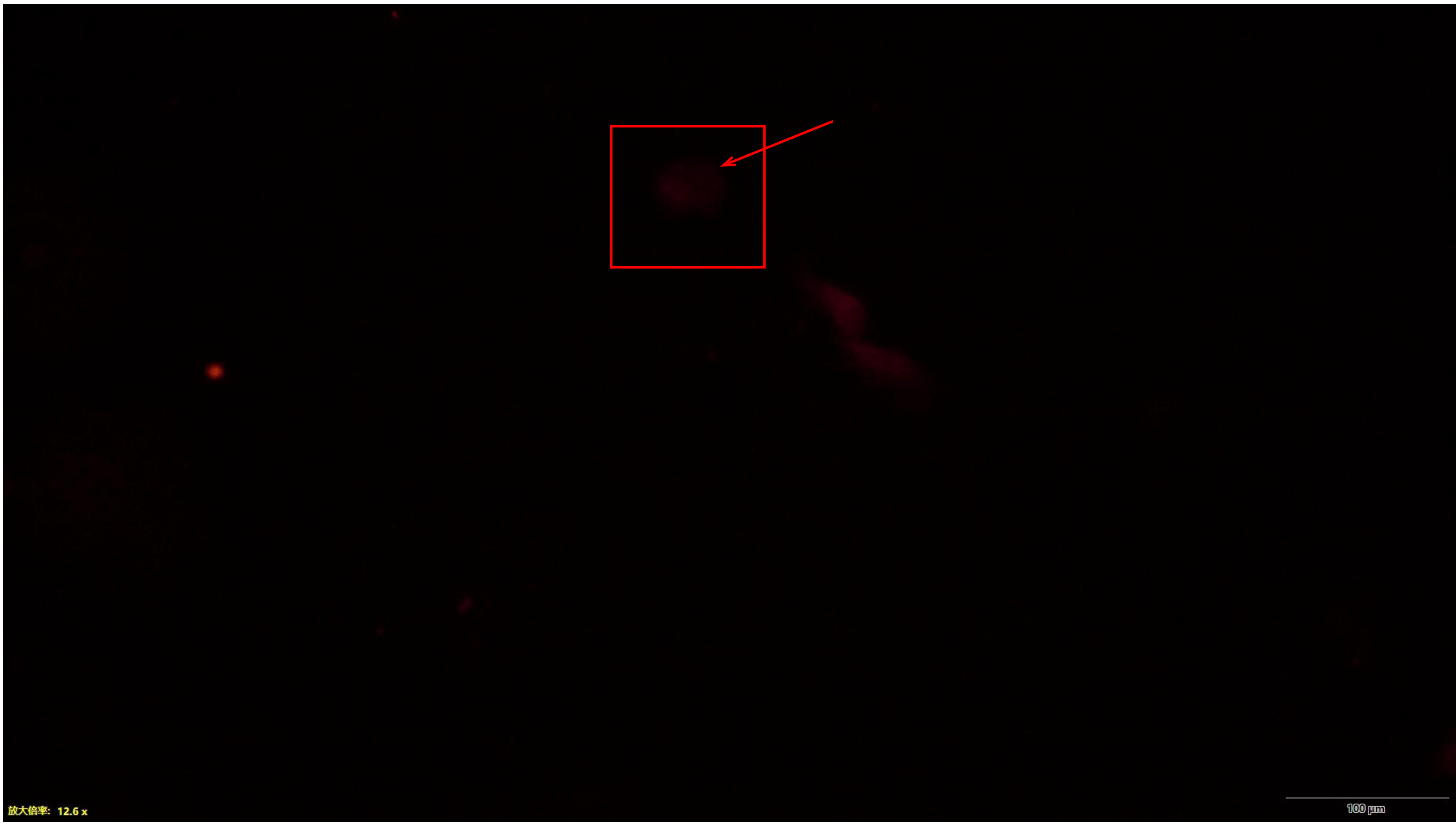

Original Image For Fig 3B
MCF-7 NC DAPI

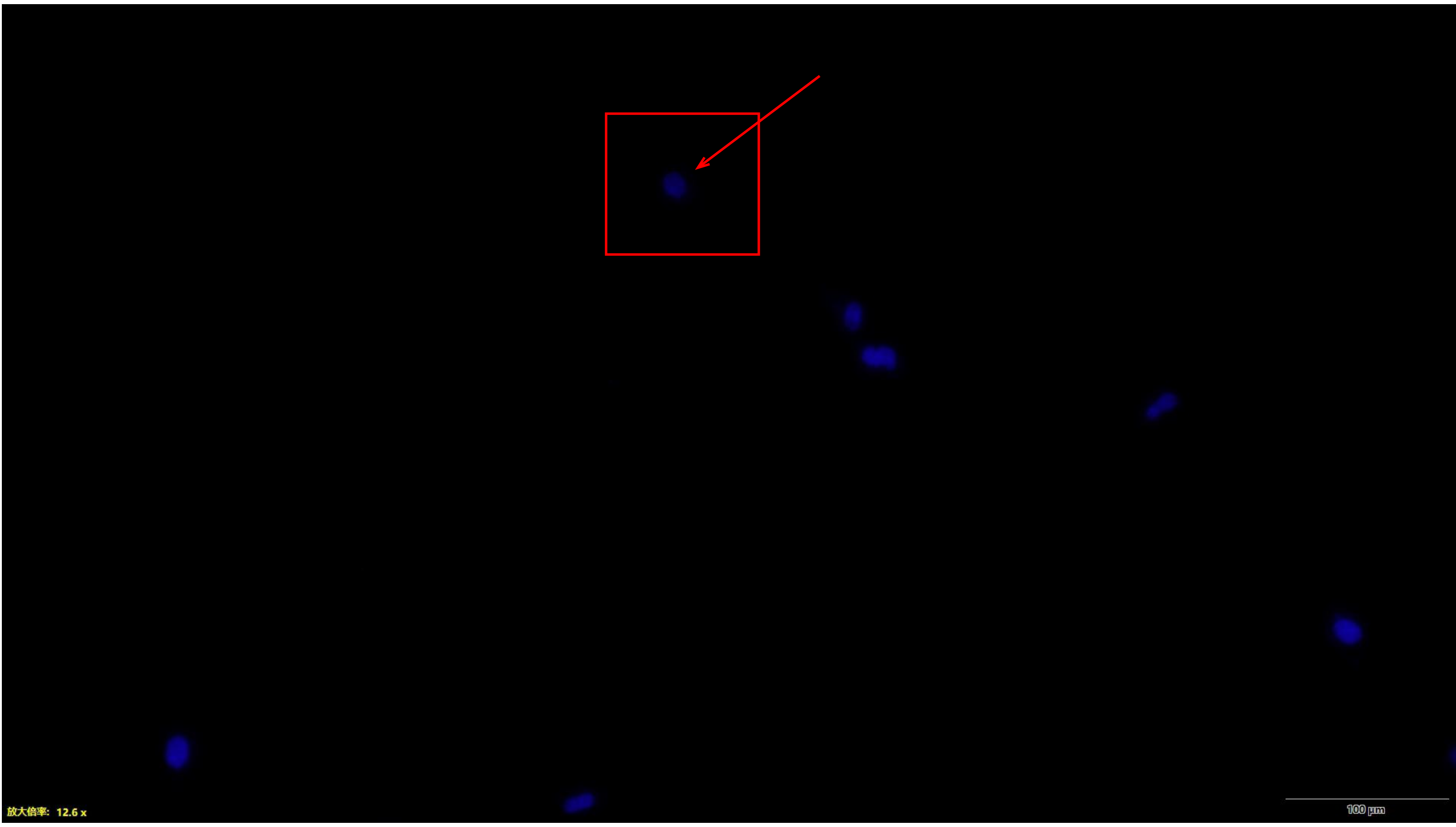


Merge

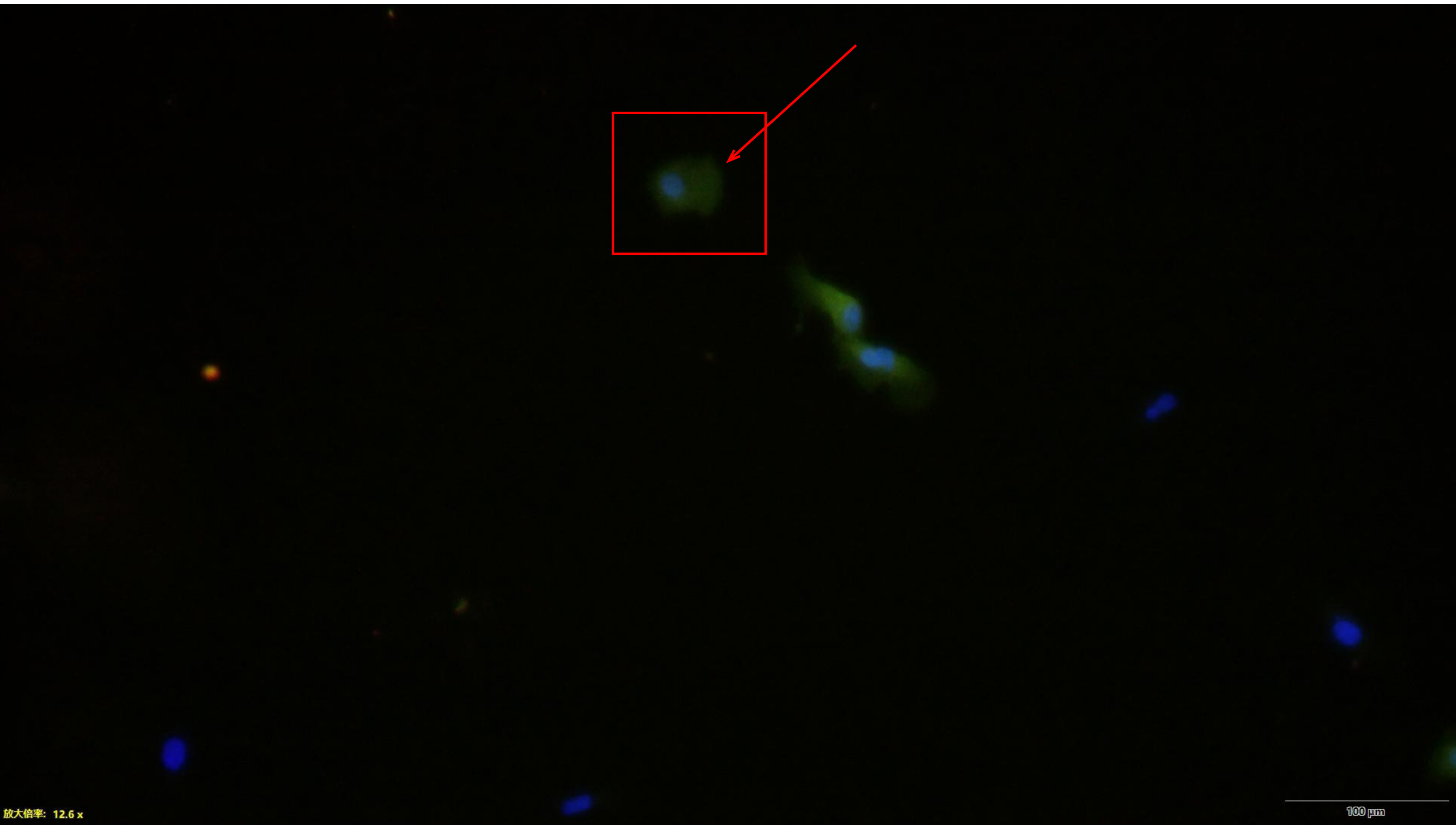

Original Image For Fig 3B
MCF-7 Treatment GFP

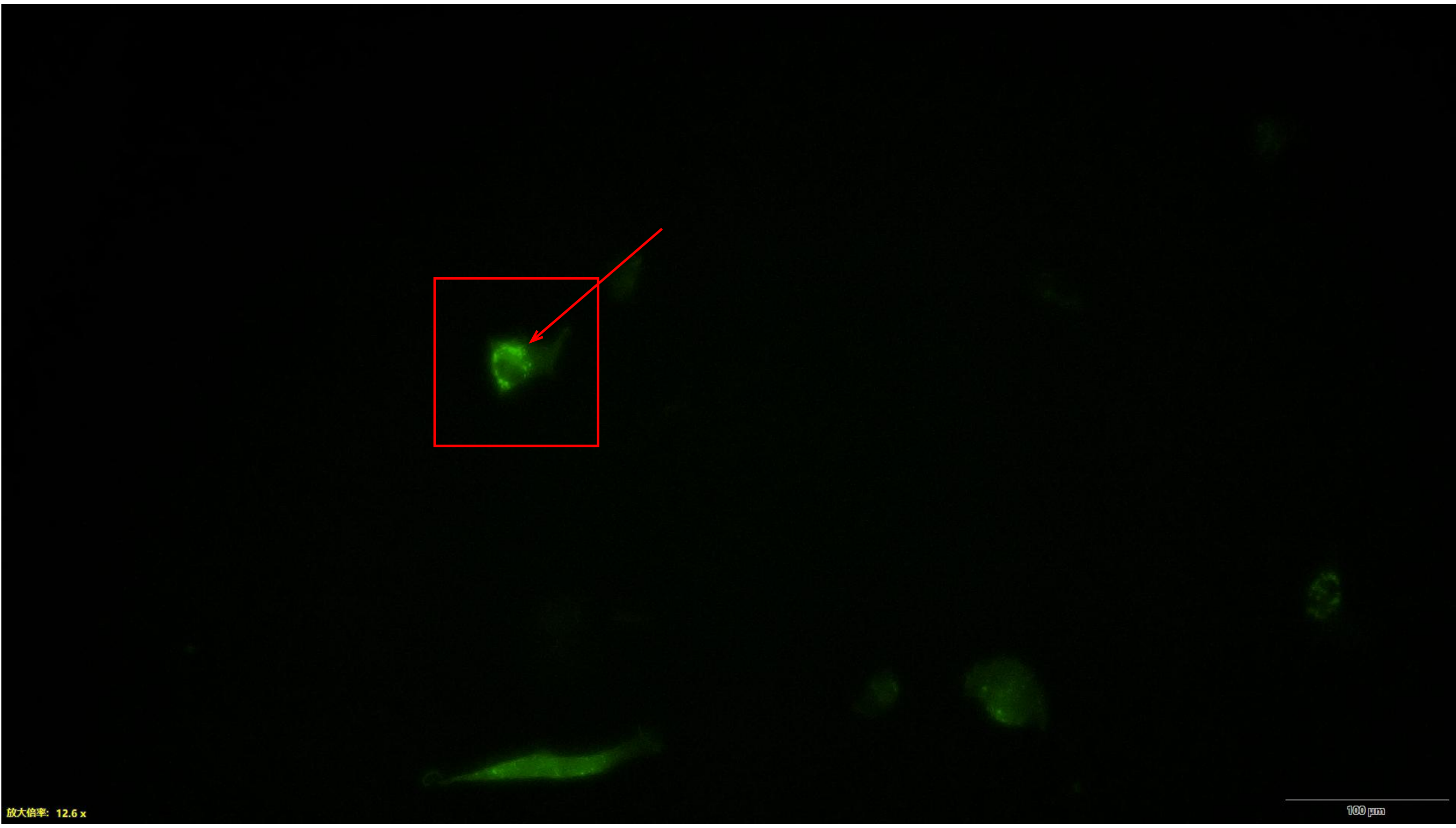


mRFP

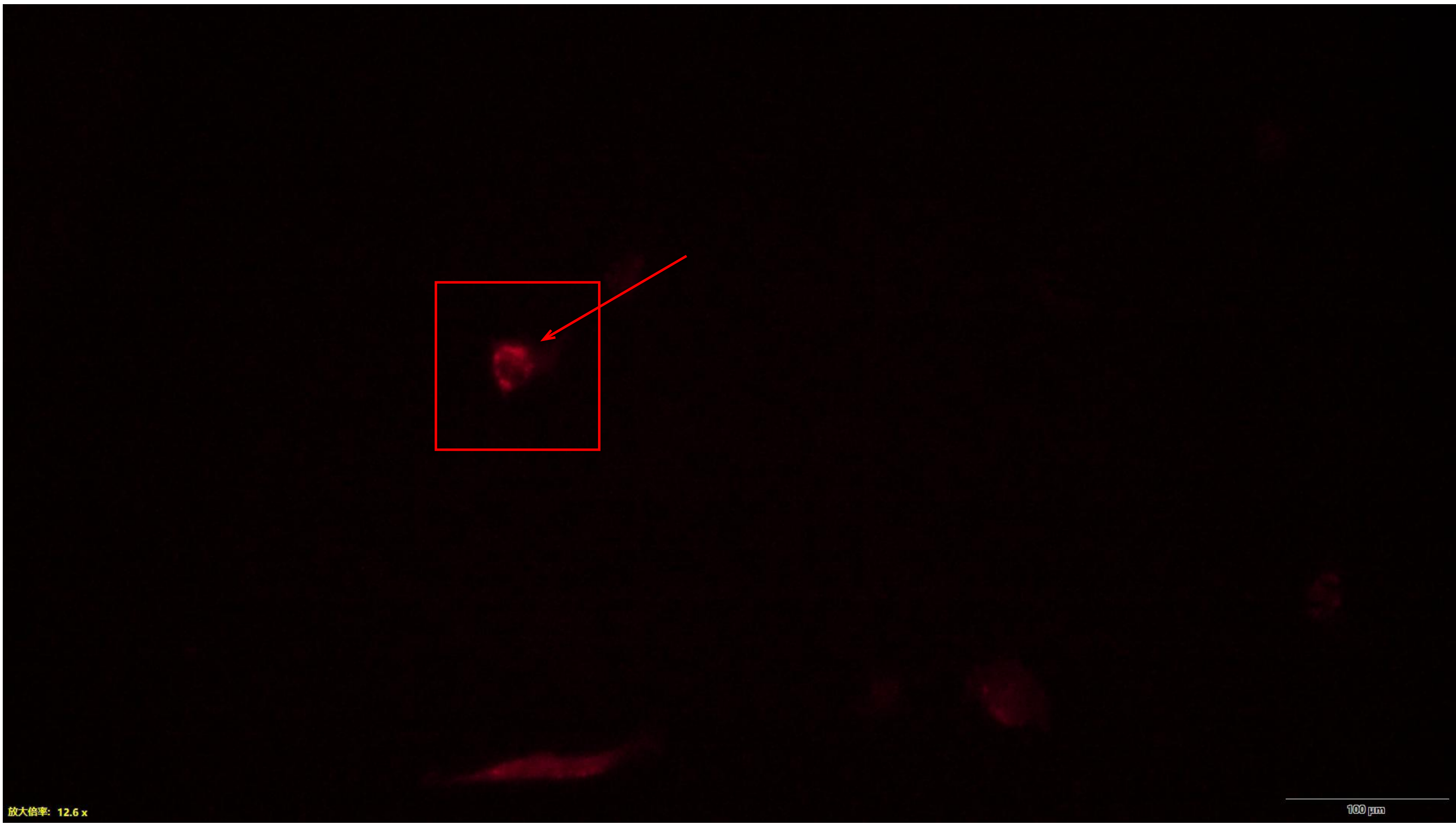

Original Image For Fig 3B
MCF-7 Treatment DAPI

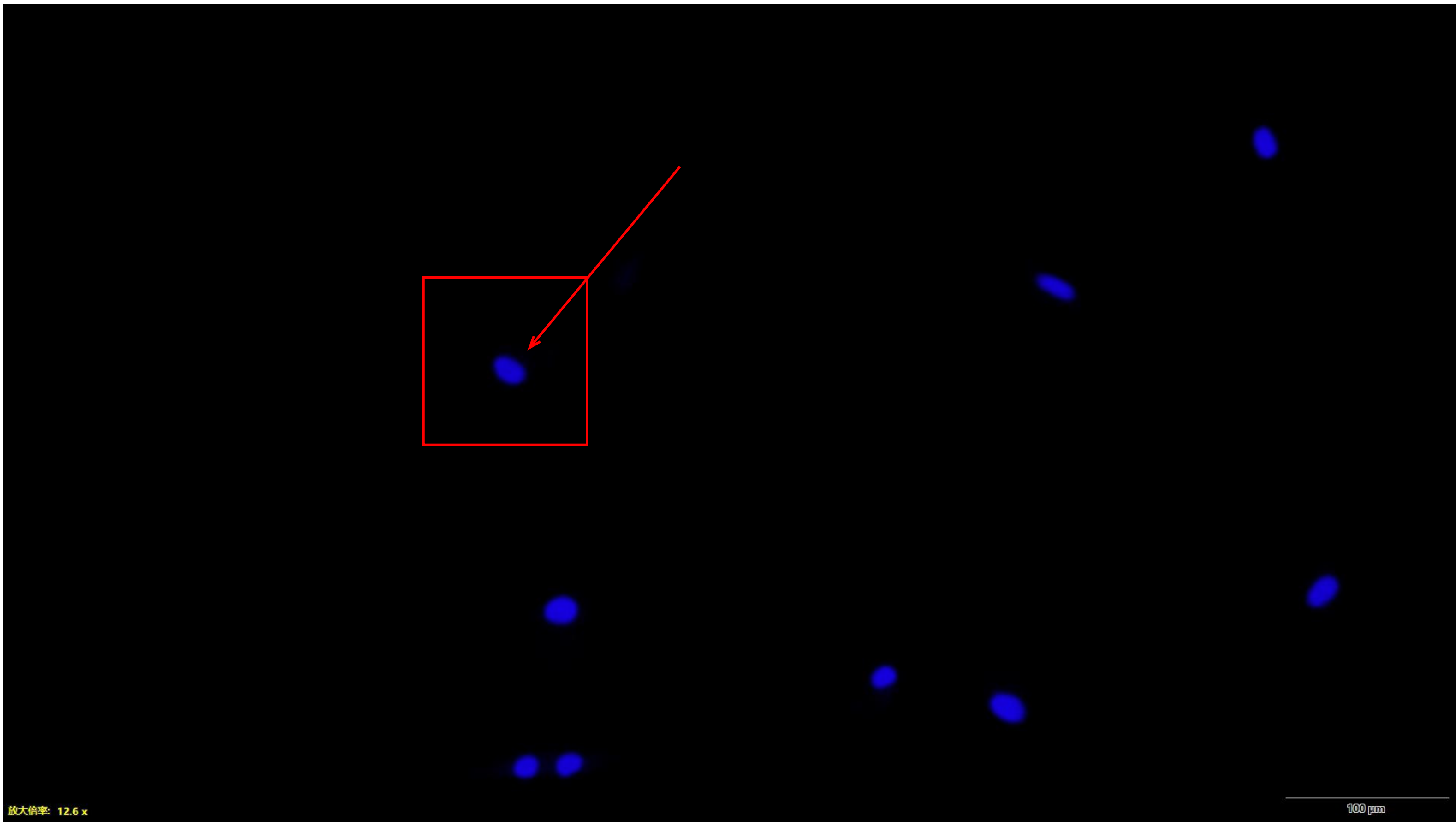


Merge

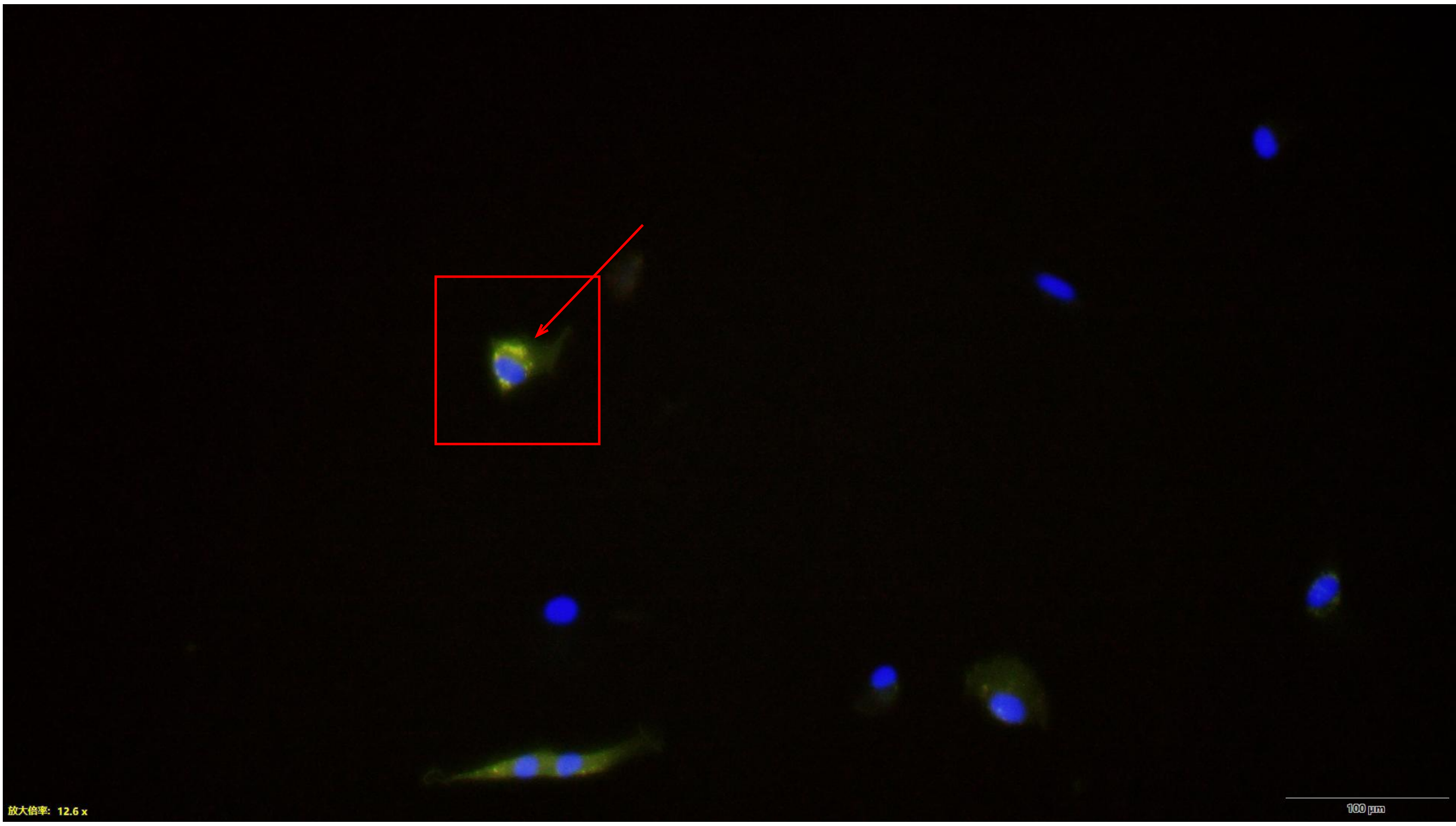

Original Image For Fig 3C MDA-MB-231

Atg5

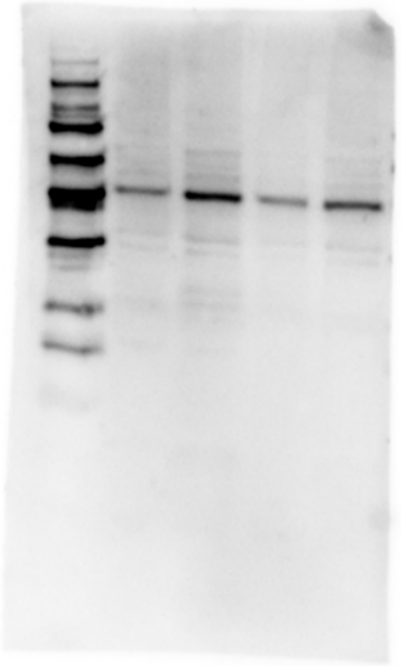

LC3

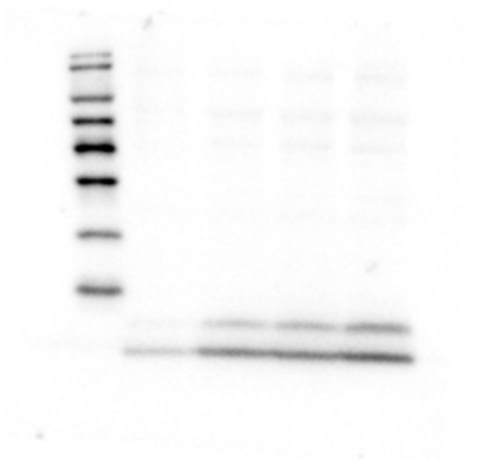

p62

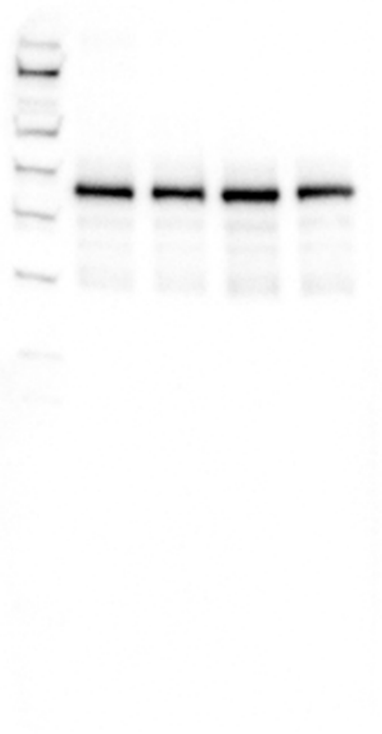

β-Tubulin

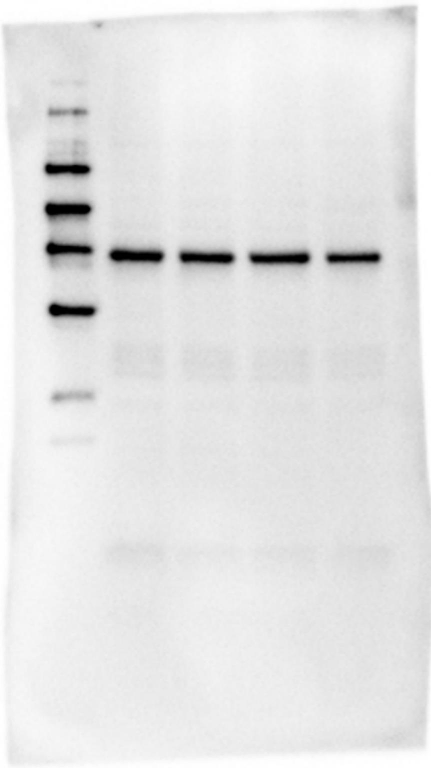

Original Image For Fig 3C MCF-7

Atg5

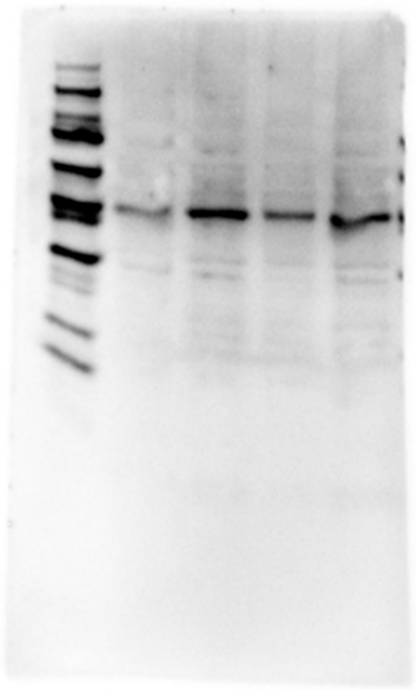

LC3

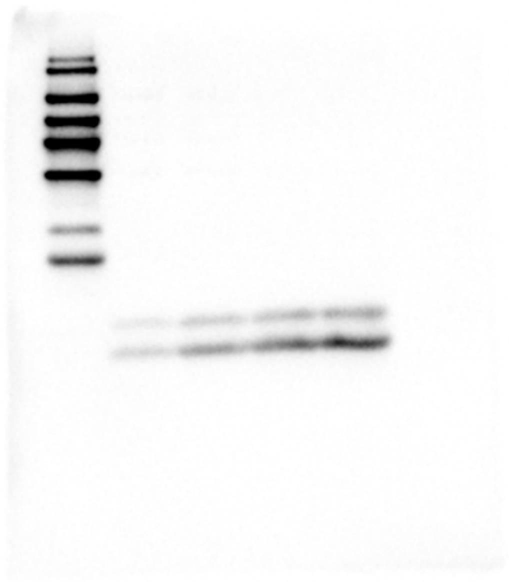

p62

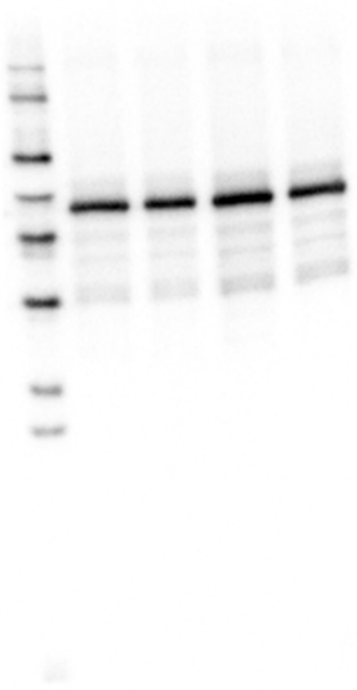

β -Tubulin

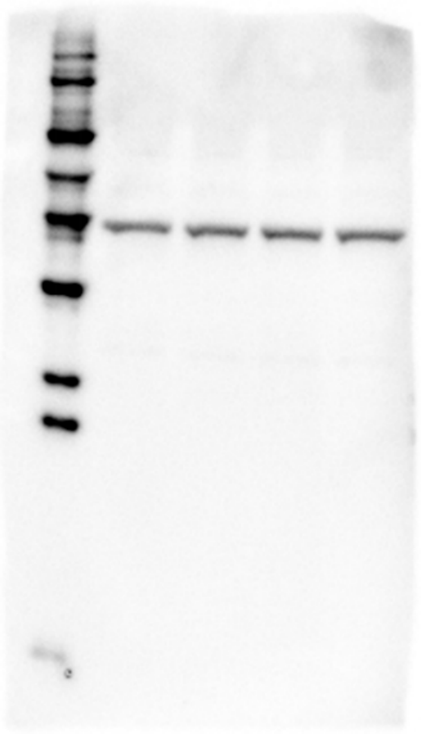

Original Image For Fig 4A
MDA-MB-231  Atg5

β-Tubulin

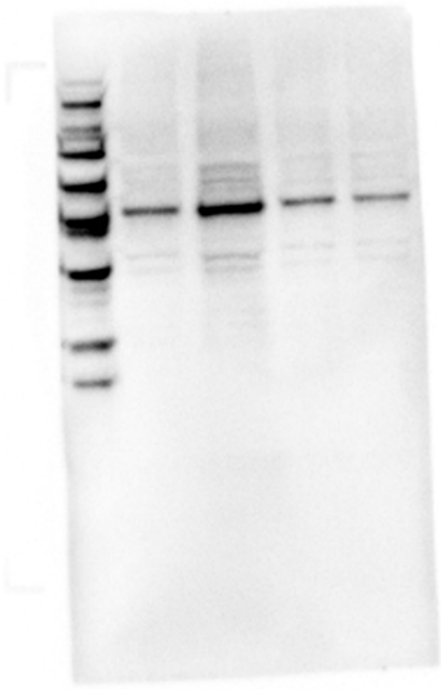

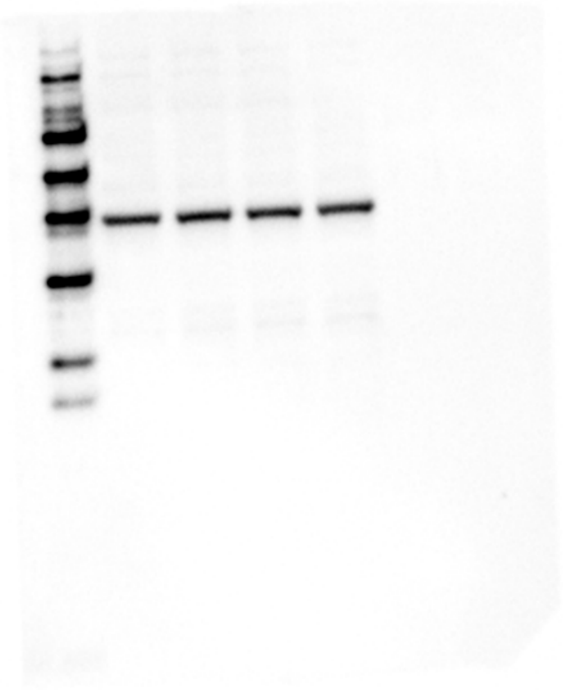

Original Image For Fig 4A
MCF-7  Atg5

β-Tubulin

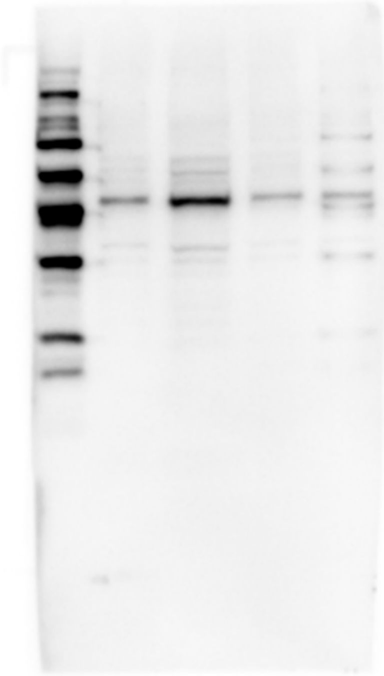

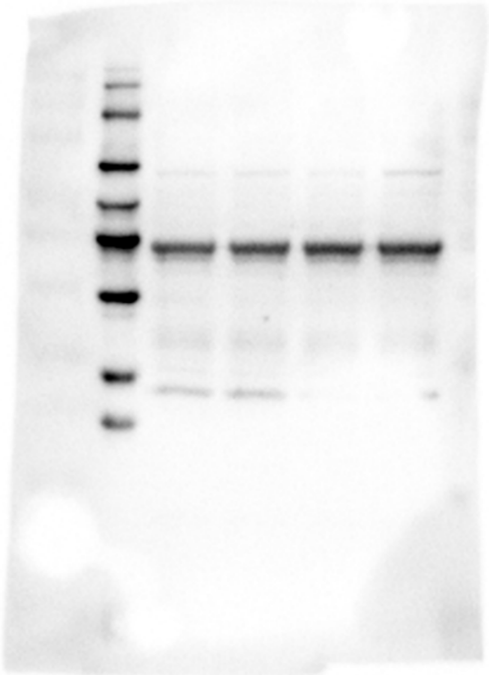

Original Image For Fig 4B MDA-MB-231

Cyclin B1

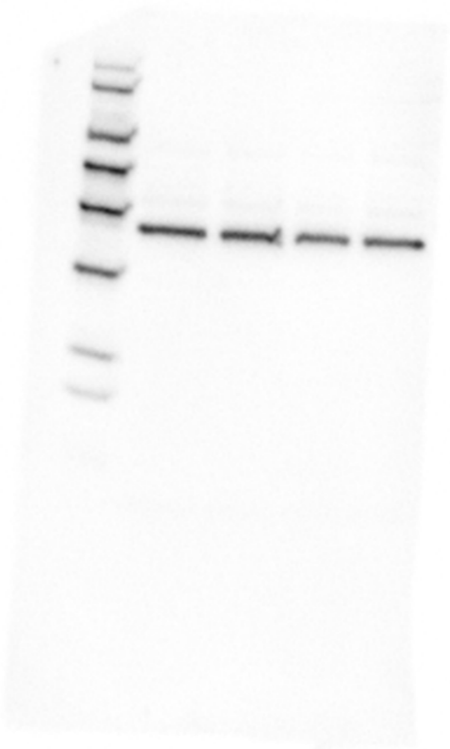

p21

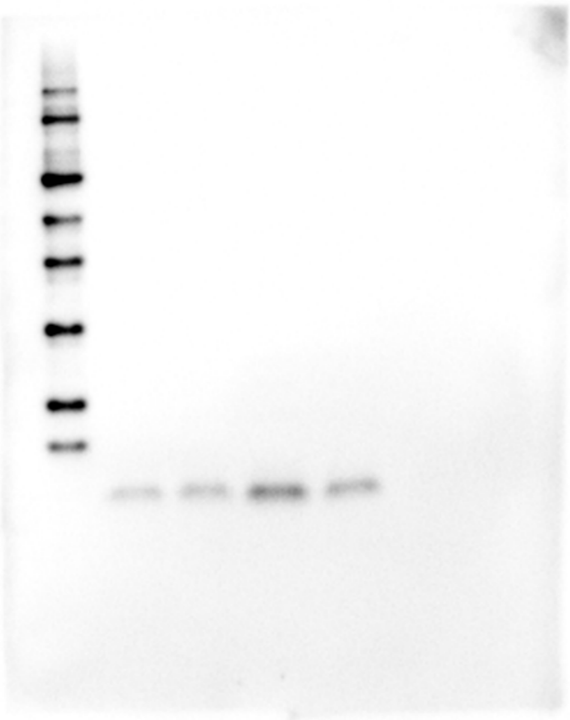

p27

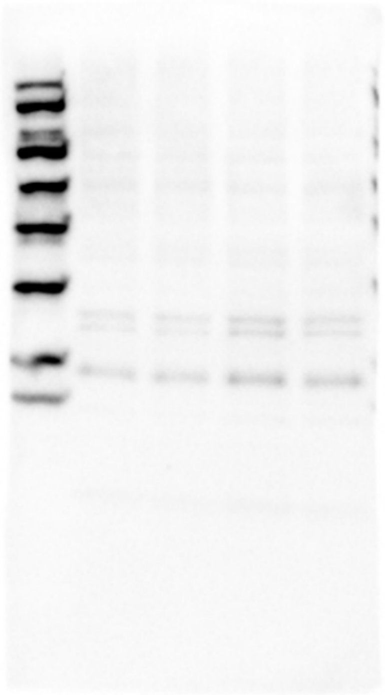

β-Tubulin

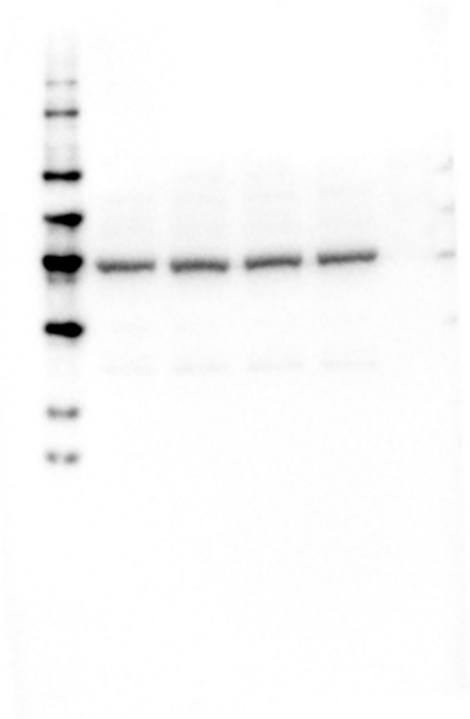

Original Image For Fig 4B MCF-7

Cyclin B1

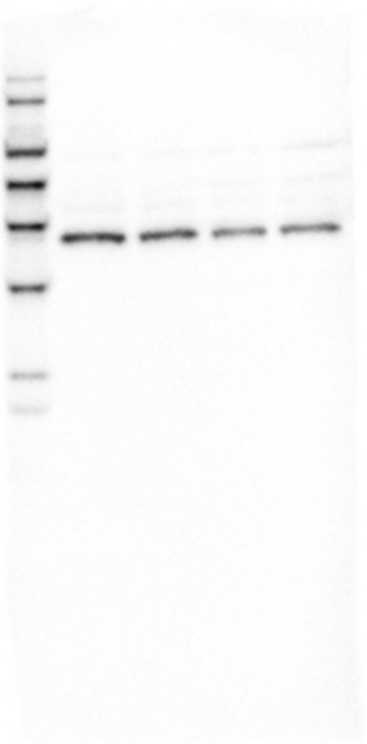

p21

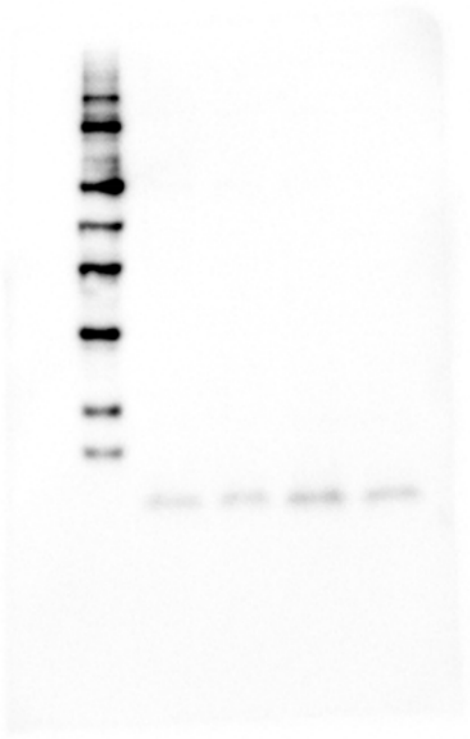

p27

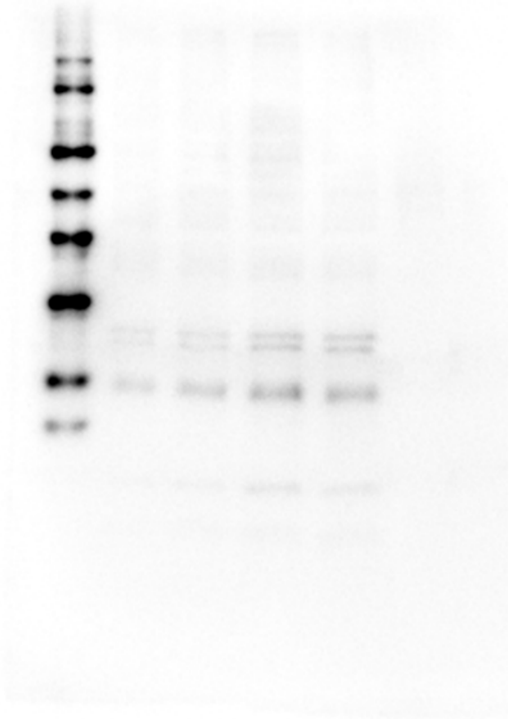

β-Tubulin

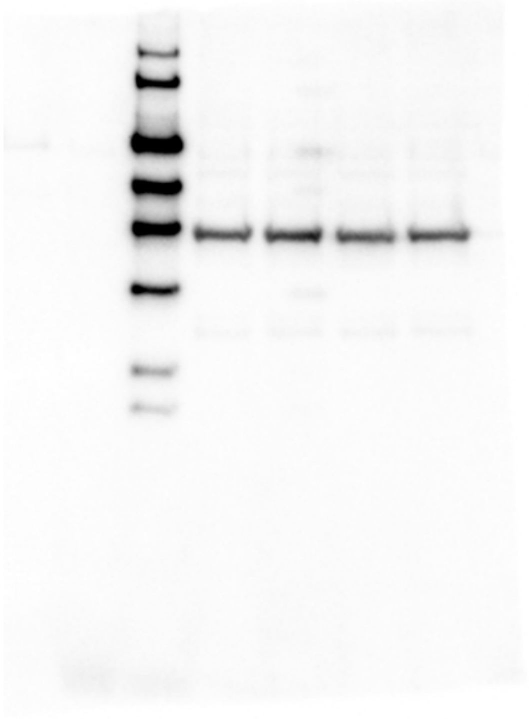

Original Image For Fig 5A MDA-MB-231

p38

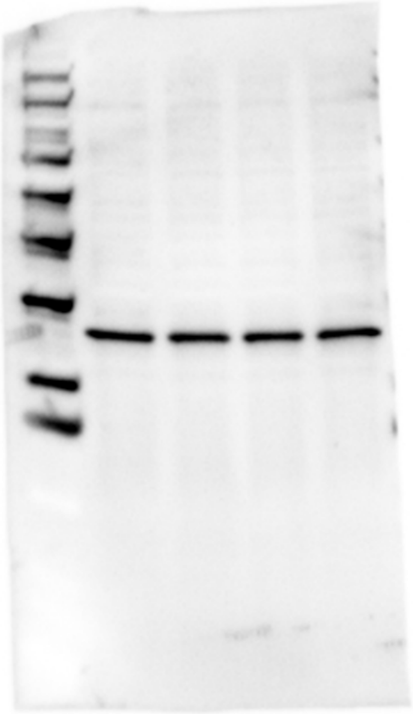

p-p38

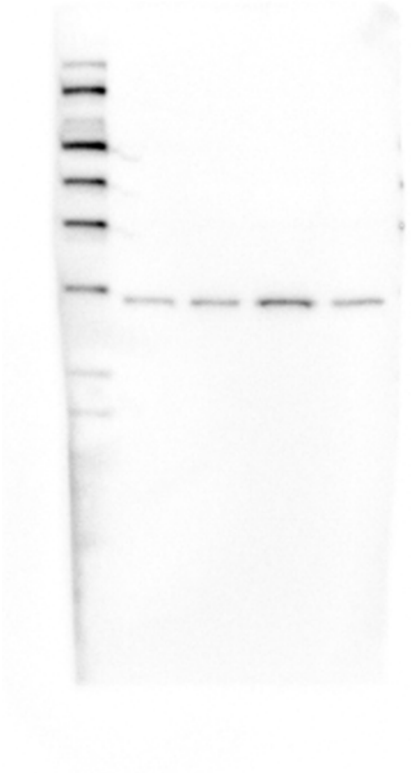

ERK1/2

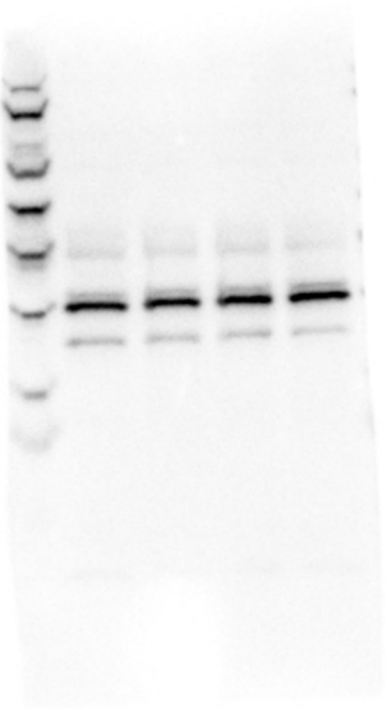

p-ERK1/2

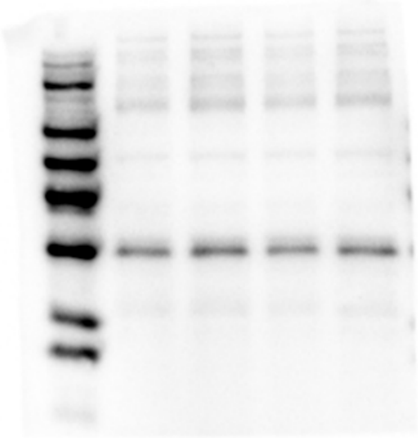

JNK

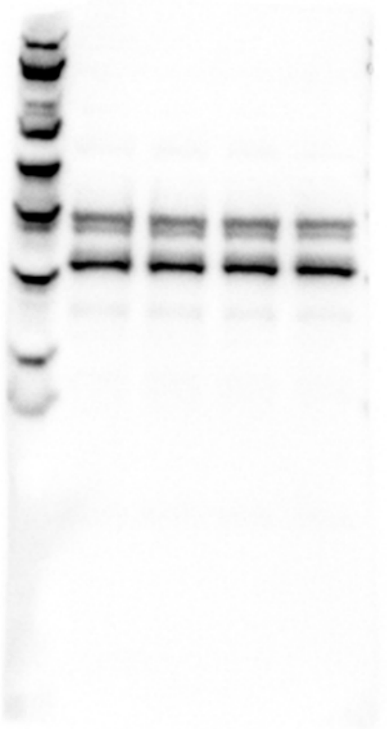

p-JNK

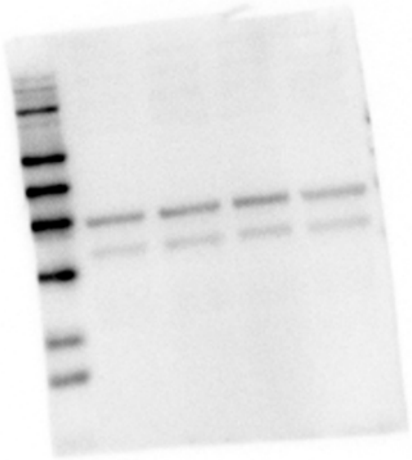

Original Image For Fig 5A MCF-7
p38

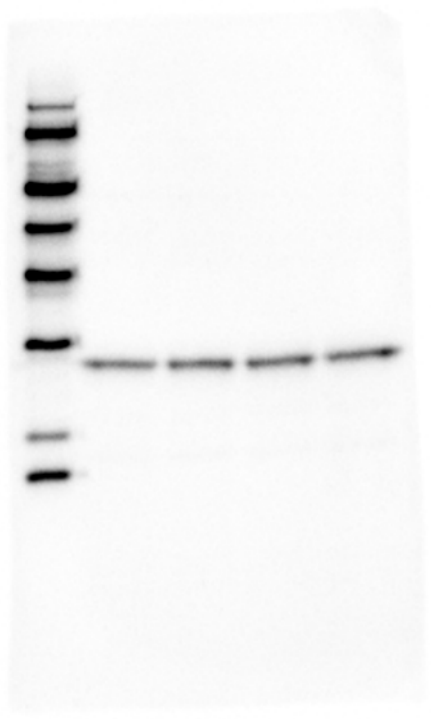

p-p38

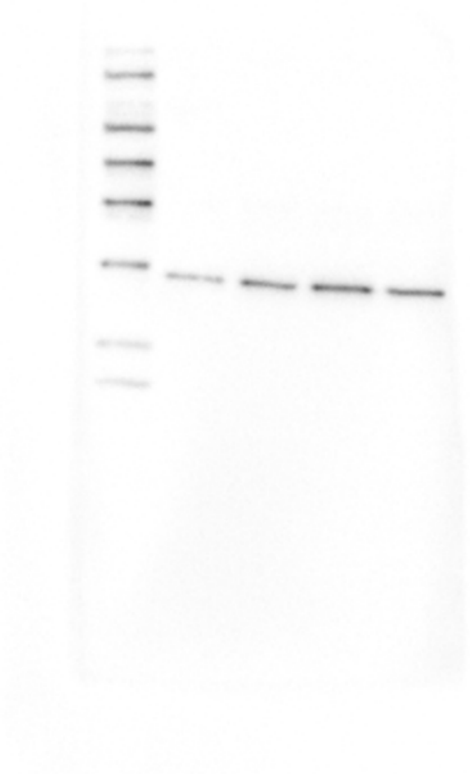

ERK1/2

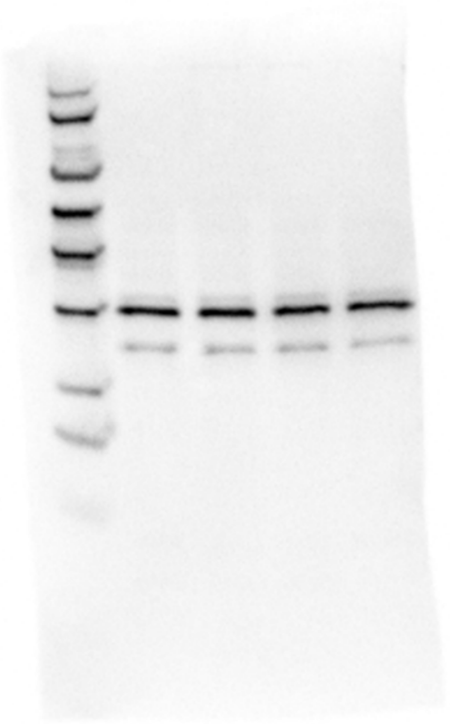

p-ERK1/2

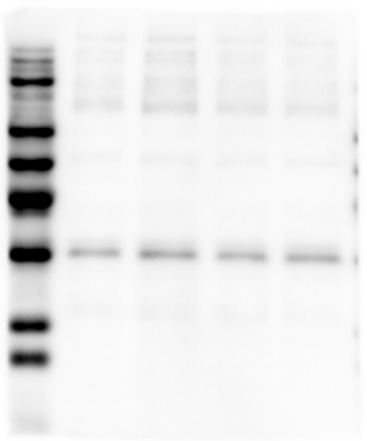

JNK

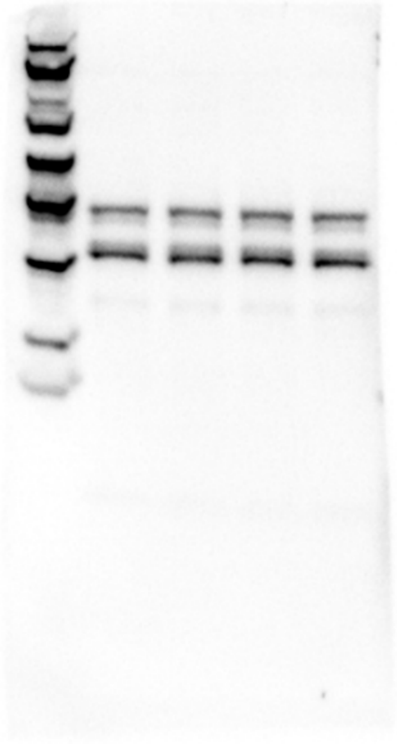

p-JNK

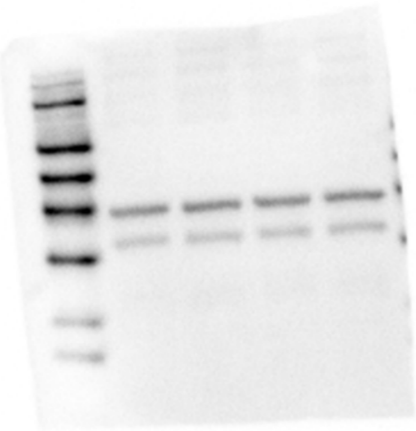

Original Image For Fig 5B
MDA-MB-231

Cyclin B1

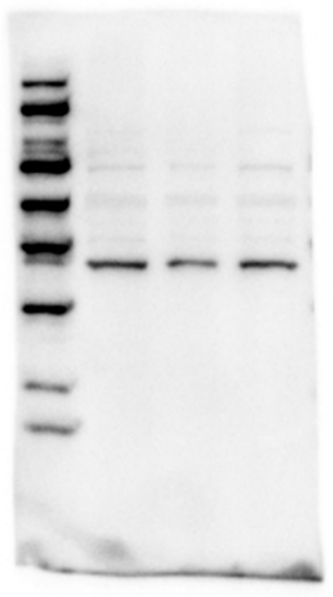

p21

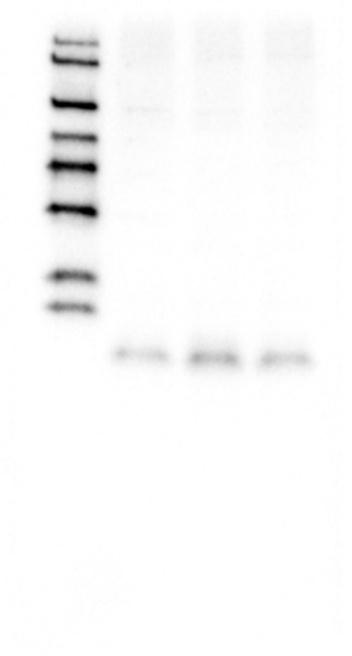

p27

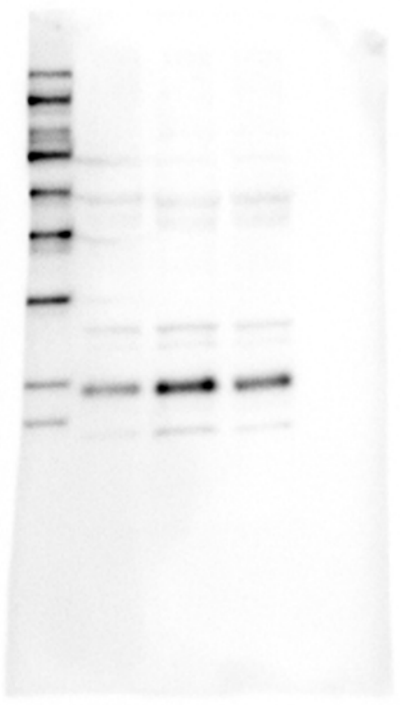

p-p38

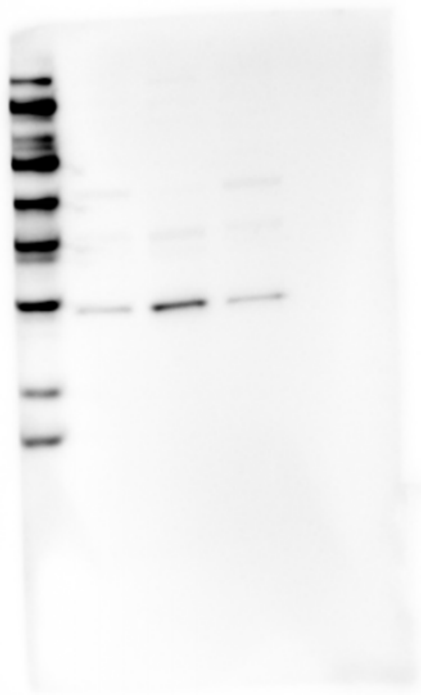

LC3

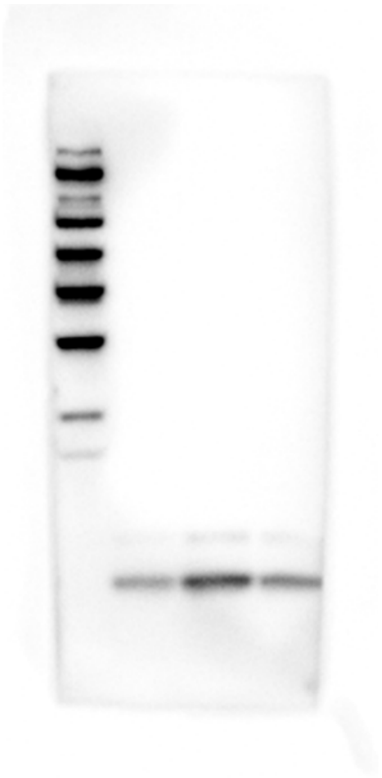

β -Tubulin

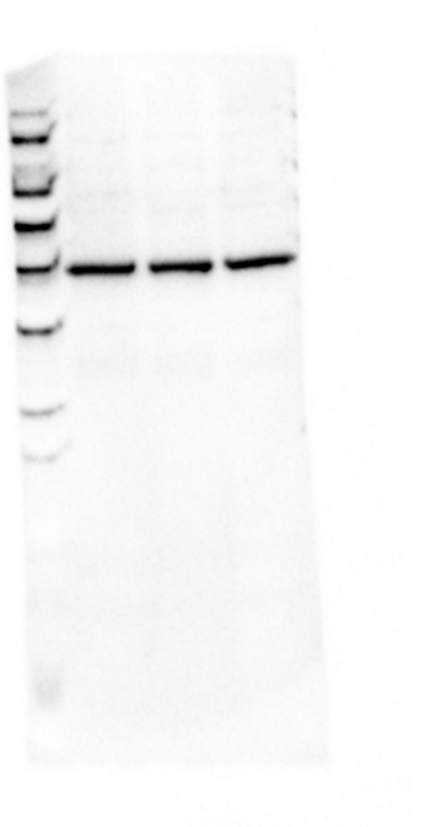

Original Image For Fig 5B
MCF-7

Cyclin B1

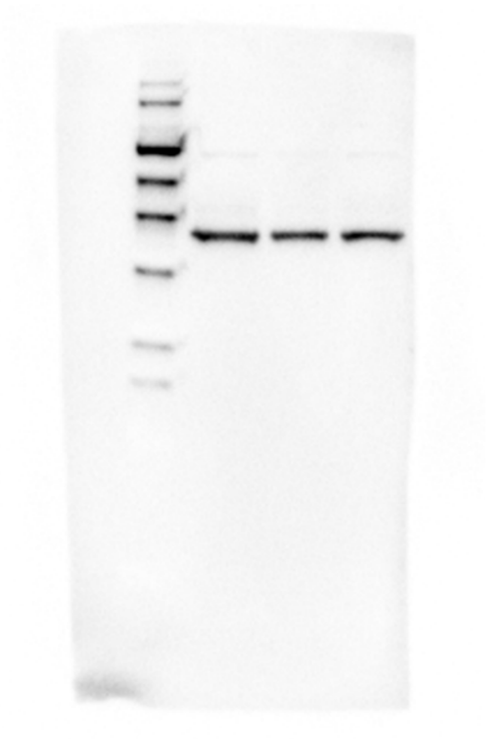

p21

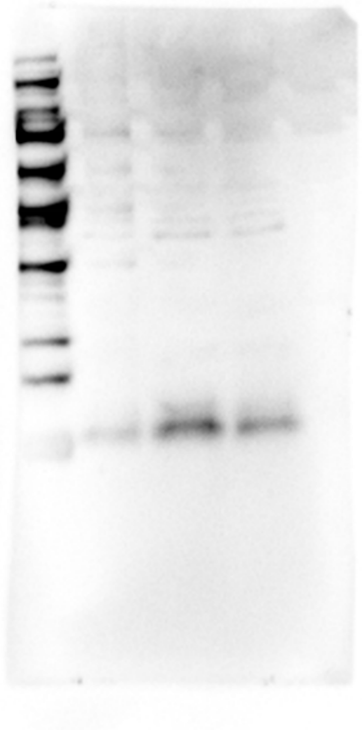

p27

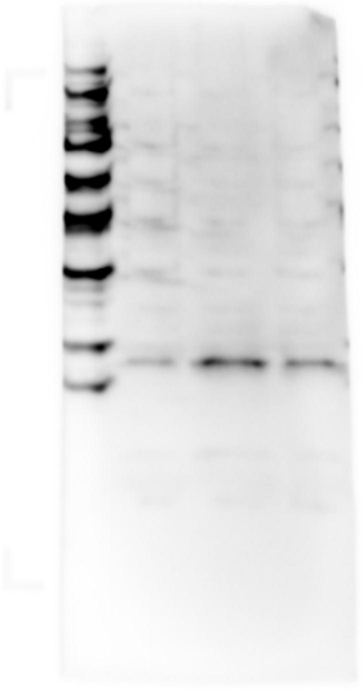

p-p38

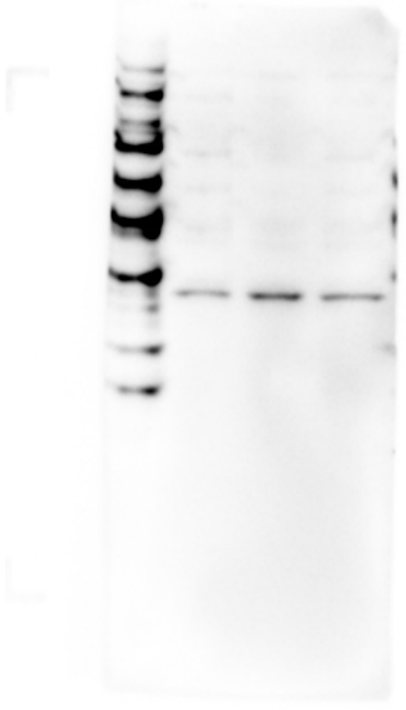

LC3

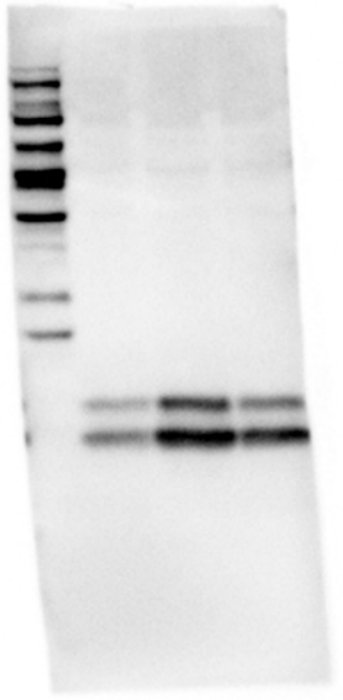

β-Tubulin

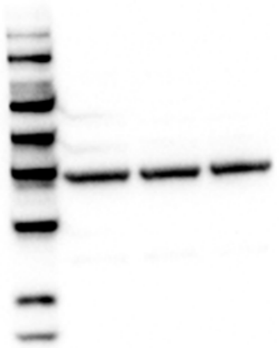

Original Image For Fig 6B

p-p38

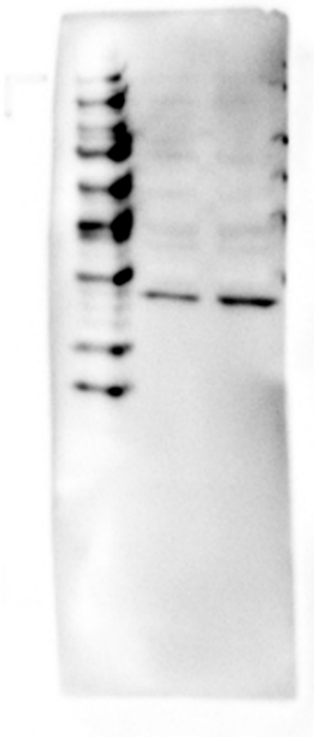

Atg5

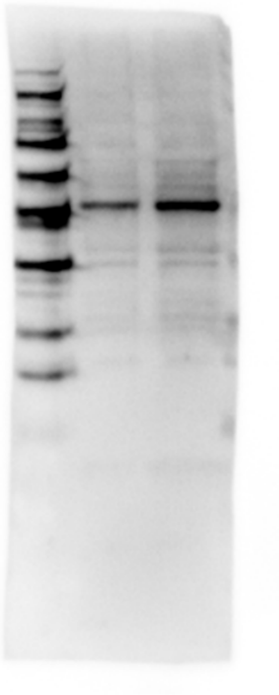

LC3

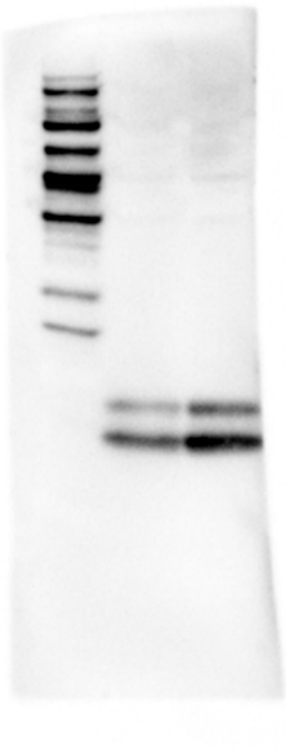

β-Tubulin

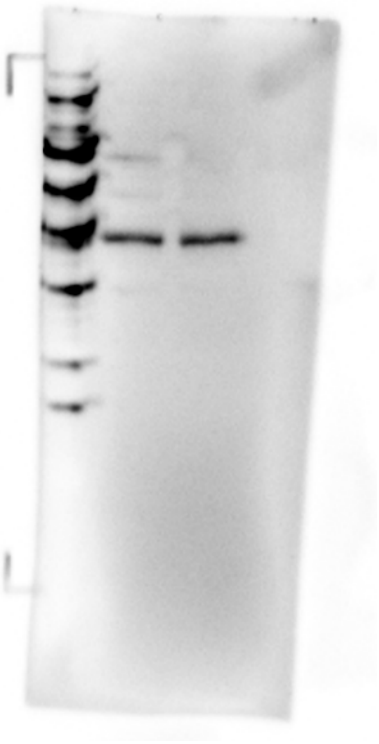

Supplement: Supplementary file 1 — Supplementary Material 1. [file 12885_2024_12293_MOESM1_ESM.pdf]
